# Innovative Poly(lactic Acid) Blends: Exploring the Impact of the Diverse Chemical Architectures from Itaconic Acid

**DOI:** 10.3390/polym16192780

**Published:** 2024-09-30

**Authors:** Miriam Carrasco-Fernández, Erika Ivonne López-Martínez, Sergio Gabriel Flores-Gallardo, Iván Alziri Estrada-Moreno, Mónica Elvira Mendoza-Duarte, Alejandro Vega-Rios

**Affiliations:** Centro de Investigación en Materiales Avanzados, SC (CIMAV), Av. Miguel de Cervantes #120, Chihuahua 31136, Chih., Mexico; miriam.carrasco@cimav.edu.mx (M.C.-F.); sergio.flores@cimav.edu.mx (S.G.F.-G.); ivan.estrada@cimav.edu.mx (I.A.E.-M.)

**Keywords:** PLA blends, itaconic acid architectures, poly(itaconic acid), itaconate copolymer, crosslinking, mechanical rheological and thermal

## Abstract

Environment-friendly polymer blends of poly(lactic acid) (PLA) and itaconic acid (IA), poly(itaconic acid) (PIA), poly(itaconic acid)-*co*-poly(methyl itaconate) (Cop-IA), and *net-*poly(itaconic acid)-*ν*-triethylene glycol dimethacrylate (Net-IA) were performed via melt blending. The compositions studied were 0.1, 1, 3, and 10 wt% of the diverse chemical architectures. The research aims to study and understand the effect of IA and its different architectures on the mechanical, rheological, and thermal properties of PLA. The PLA/IA, PLA/PIA, PLA/Cop-IA, and PLA/Net-IA blends were characterized by dynamic mechanical thermal analysis, rotational rheometer (RR), thermogravimetric analysis, differential scanning calorimetry, X-ray diffraction, and scanning electron microscopy. The complex viscosity, storage module, and loss module for the RR properties were observed in the following order: PLA/Cop-IA, PLA/Net-IA, and PLA/PIA > PLA > PLA/IA. Thermal stability improved with increasing concentrations of Cop-IA and Net-IA. In the same way, the mechanical properties were enhanced. In addition, the micrographs illustrated the formation of fibrillar structures for all blends. The crystallinity degree displayed higher values for the blends that contain Net-IA > Cop-IA than IA > PIA. Therefore, IA and its architectures can influence these studied properties, which have potential applications in disposable food packing.

## 1. Introduction

The generation of polymer blends with poly(lactic acid) (PLA) via melt blending is a strategy that has improved its drawbacks, such as elasticity, impact resistance, thermal stability, heat resistance, rheological properties, and biodegradability over the years [1]. For example, Liu et al. reported that the PLA/Poly(Butylene adipate-*co*-terephthalate) (PBAT)/Graphene blend (20:79.8:0.2 wt.%) increased by 24%, 5%, and 62% compared with neat PLA in the tensile strength, elongation at break, and Young’s modulus, respectively. The elongation at break, elastic modulus, and tensile strength reached 87.3%, 3.8 GPa, and 75.6 MPa, respectively, for the PLA/CNTs/MMT blend (98.5:0.5:1 wt%) [2]. The mixture of PLA with graphene oxide dispersed in epoxidized soybean oil and then crosslinked with sebacic acid to a concentration of 80:20 (wt %) has antistatic properties and shape memory foam [3]. Li et al. reported PLA/Poly(hydroxybutyrate) (PHB) blends where the impact strength, flexural modulus, and Young’s modulus increased by 80.5%, 16.4%, and 41.4%, respectively, for PLA/PHB 80:20 wt.% compared with neat PLA [4].

Additionally, most research is focused on mixing PLA with another biobased or synthetic polymer. The architecture typically used is a homopolymer, e.g., PLA/Polycaprolactone(PCL) [3,5], PLA/Polyethylene glycol (PEG) [6,7,8,9,10], PLA/PHB [4], PLA/Polyethylene terephthalate(PET) [11,12], PLA/Polyamide (PA) [13,14,15,16], PLA/PBAT [17,18,19,20], PLA/starch [21,22,23], or PLA/Polyvinyl alcohol (PVA) [24]. The applications of these polymer blends are used in various fields, such as flexible packaging [6,22,24], engineering [16], 3D printing [17,18], medical devices (e.g., sutures, medical implants, catheters) [15,25], shape memory materials [26], coating [7], cushioning application [20], and agricultural films [15].

Regarding mixtures of PLA with copolymers or complex architectures, there are some works in the literature. For instance, Odent et al. reported PLA/poly(ε-caprolactone-*co*-D-L-lactide) blends. The blends exhibited increased impact strength, correlated with the presence of ribbon-like rubbery microdomains [27]. Li et al. reported PLA/P(3HB-*co*-4HB) blends. The blends exhibit improved mechanical properties and an increased crystallinity degree in the materials [4]. Therefore, the study of PLA/bioplastics blends, particularly with itaconate copolymers and crosslinking networks of itaconic acid (IA), has been relatively unexplored.

Furthermore, IA is generally utilized as a versatile building block for synthesizing complex architecture renewable sources [28]. Krishnan et al. synthesized by polycondensation a bioelastomer (IA, sebacic acid, 1,4 butanediol, and lactic acid)-based aliphatic copolyester elastomer, which was blended with PLA in the presence of free radical initiator dicumyl peroxide [29]. Ivorra-Martinez et al. reported mixtures of PLA/dibutyl itaconate (80:20 wt.%) by reactive extrusion. The blend considerably reduces the T_g_, exhibiting a plasticization effect. The Young’s modulus, tensile strength, and elongation at break reached 108 MPa, 20.6 MPa, and 209%, respectively [30]. For this reason, our research utilizes IA to obtain different architectures because, in addition to being a green monomer, it has different reactive functional groups, in particular alkene and carboxylic acid.

In contrast, grafted polymer synthesis through the functionalization of PLA with IA has been extensively studied [31]. Agustin-Salazar et al. reported that a blend of PLA and pecan nutshell grafted with IA increased the thermal stability and enhanced the crystallization phenomenon. Additionally, the blend exhibited a strain of 11.7% at 140 °C [31]. Walallavita et al. reported a blend of itaconic anhydride grafted with PLA/Novatein (50:50 wt%), with the tensile strength and impact strength increasing by 42% and 36%, respectively [32]. Kučera et al. reported that itaconic anhydride grafted with PLA in melt blending achieved a grafting efficiency of about 60% [33]. The graft copolymer architecture in our research was not explored due to these works at this time.

Despite these investigations, few studies analyze the effect of polymer architecture on PLA, and even fewer studies on IA. Here, environment-friendly polymer blends of poly(lactic acid) (PLA) and IA, poly(itaconic acid) (PIA), poly(itaconic acid)-*co*-poly(methyl itaconate) (Cop-IA), and *net-*poly(itaconic acid)-*ν-*triethylene glycol dimethacrylate (Net-IA) were developed via melt blending. The research aims to study and understand the effects of IA and its different architectures on the mechanical, rheological, and thermal properties of PLA. Furthermore, the properties of the PLA/IA, PLA/PIA, PLA/Cop-IA, and PLA/Net-IA blends, including rheological, morphological, thermal, mechanical, and thermomechanical properties, were studied in detail. Likewise, the architectures obtained from IA were analyzed, establishing their effects on the mixtures with PLA.

## 2. Materials and Methods

### 2.1. Materials

Poly(lactic acid) (2003D) (PLA) with 4.3% mol of D was supplied by NatureWorks (Minneapolis, MN, USA), with a density of 1.24 g/cm^3^ and a melt index of 6 g/10 min (210 °C). Itaconic acid (IA), potassium persulfate (KPS), *p*-toluenesulfonic acid, and triethylene glycol dimethacrylate (TEGDMA) were acquired from Sigma Aldrich (Burlington, MA, USA). In addition, methanol, acetone, and distilled water were acquired from CTR Scientific (Chihuahua, Chih., Mexico).

### 2.2. Synthesis of Itaconic Acid Architectures

Poly(itaconic acid) (PIA) was synthesized via radical polymerization according to the methodology reported by Nagai and Yoshida [34]. The polymerization time was 50 h at a temperature of 75 °C with constant mechanical stirring at 120 rpm. The homopolymer was then purified by washing (three times) with distilled water and acetone. Finally, it was dried at room temperature.

The copolymer (poly(itaconic acid)-*co-*poly(methyl itaconate)) (Cop-IA) was prepared from the esterification of (PIA) (1 g) with methanol (2 mol), using the catalyst *p*-toluenesulfonic acid (5 wt%). The substances were added to a reactor (250 mL) equipped with a reflux system and constant magnetic stirring. The synthesis was kept at 80 °C for 4 h. The copolymer was filtered and washed with distilled water. Finally, the product was dried at room temperature.

The synthesis of *net-*poly(itaconic acid)-*ν*-triethylene glycol dimethacrylate (Net-IA) was carried out via radical polymerization, specifically in a reactor (1 L) equipped with a magnetic stirred system. Then, 1.5 mol of IA, 0.05 mol of KPS, 0.5 mol of TEGDMA, and 1 L of distilled water were added. The polymerization system was maintained with constant stirring at 150 rpm at 70 °C for 3 h. Finally, the synthesis product was filtered, washed, and dried at room temperature.

### 2.3. Preparation of Blends of PLA/Itaconic Acid and Its Architectures

Prior to blending and formulating, the PLA resin was vacuum-dried at 65 °C in a Fisher Scientific vacuum oven for 12 h. Next, the PLA/IA, PLA/PIA, PLA/Cop-IA, and PLA/Net-IA blends were prepared in concentrations of 0.1, 1, 3, and 10 wt %. The formulations were mixed in a Brabender internal mixer, model DDRV501 (Brabender Instruments Inc., South Hackensack, NJ, USA), at a temperature of 180 °C, a speed of 60 rpm, and a total mixing time of 8 min, employing CAM-type blades.

### 2.4. Film Preparation

The films were prepared by hot compression molding (Carver Inc., Wabash, IN, USA). First, the mixtures obtained were ground using a blade grinder (Fritsch model Pulverisette, Pittsboro, NC, USA). Next, the ground samples were molded at 165 °C for 3 min without applying pressure. Afterward, 3 tons of pressure was applied for 4 min. Finally, the samples were cooled down to 25 °C by recirculating water.

### 2.5. Characterization

#### 2.5.1. Fourier Transform Infrared Spectroscopy

The PLA blends, IA, and its architectures were analyzed using an IR Affinity 1S B spectrometer equipped with a diamond ATR accessory (Shimadzu, Kyoto, Japan). The samples were measured in transmittance mode in the 4000 cm^−1^ to 400 cm^−1^ range with a resolution of 4 cm^−1^.

#### 2.5.2. Electron Microscopy

Morphological analysis was performed on continuous carbon membrane copper grids (200 mesh). IA and its diverse architectures were morphologically analyzed using a transmission electron microscope (TEM, Hitachi 7700, Tokyo, Japan) with an acceleration voltage of 40 kV. Additionally, the PLA blends were morphologically evaluated through secondary electron analysis using a scanning electron microscope (SEM, Hitachi SU3500, Tokyo, Japan) with a voltage of 10 kV, examining both the surface and cross-sectional views. In addition, the samples analyzed in the cross-sections were prepared by coating the sample with a layer of gold using a sputtering system (Denton vacuum DESK II, Moorestown, NJ, USA).

#### 2.5.3. Rheological Properties

The storage modulus and complex viscosity of the PLA/IA, PLA/PIA, PLA/Cop-IA, and PLA/Net-IA blends were evaluated using a Physica MCR 501 rotational rheometer (Anton Paar, Graz, Austria), employing parallel plate geometry. A frequency sweep was conducted in the range of 0.01–100 Hz, with a strain amplitude of 0.1%, and the reference temperature for the analysis was set at 170 °C.

#### 2.5.4. Differential Scanning Calorimetry

DSC was employed to evaluate the thermal properties and crystallinity degrees of the PLA/IA, PLA/PIA, PLA/Cop-IA, and PLA/Net-IA blends. These properties were observed during the second heating scan. The measurements were conducted using a DSC Q200 Instruments (TA Instruments, New Castle, DE, USA) under an air atmosphere (50 mL/min). The heating rate was 10 °C/min from 30 to 220 °C.

#### 2.5.5. X-ray Diffraction

For the XRD analysis of the blends, a Bruker D8 Advance diffractometer (Bruker Corp., Billerica, MA, USA) was employed. The instrument was operated with CuKα radiation (λ = 1.54 Å) over 2θ of 5° to 80°, with a step size of 0.02°.

#### 2.5.6. Dynamic Mechanical Thermal Analysis

The thermomechanical behavior of the PLA blends was evaluated using a DMA RSAIII (TA Instruments, New Castle, DE, USA) in tension mode. The samples were analyzed over a temperature ramp from 30 to 180 °C, with a heating rate of 5 °C/min. The deformation frequency was set at 1 Hz, with a strain percentage of 0.1%.

#### 2.5.7. Thermogravimetric Analysis

The thermal stability of the PLA/IA, PLA/PIA, PLA/Cop-IA, and PLA/Net-IA blends was determined using a TGA Q5000 (TA Instruments, New Castle, DE, USA). The samples were heated from 25 to 700 °C, with a heating rate of 10 °C/min under an air atmosphere (50 mL/min), and each analysis was performed in triplicate.

#### 2.5.8. Mechanical Analysis

The effect of IA and its architectures on the elasticity and elongation of the PLA blends was evaluated using a DMA RSAIII (TA Instruments, New Castle, DE, USA) in tension mode. In the stress-deformation test, a constant deformation rate of 0.01 mm/s at 25 °C was applied.

## 3. Results and Discussion

**Scheme 1:**
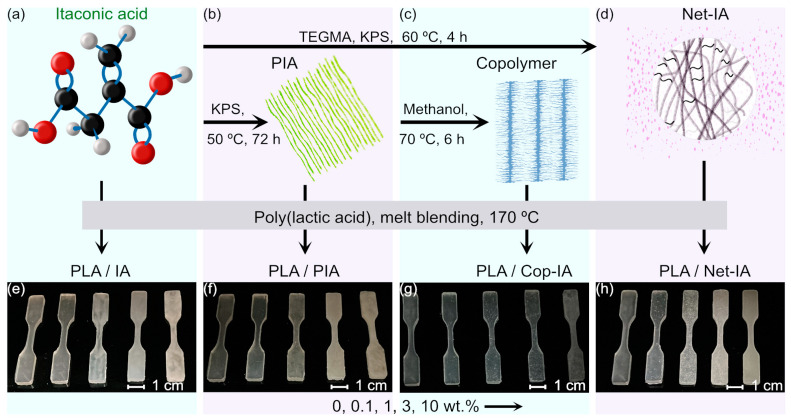
(**a**) Itaconic acid (IA) monomer; (**b**) polymerization of IA, poly(itaconic acid) (PIA); (**c**) PIA esterification with methanol, poly(itaconic acid)-co-poly(methyl itaconate) (Cop-IA); (**d**) crosslinking IA with triethylene glycol dimethacrylate (TEGMA), net-poly(itaconic acid)-n-triethylene glycol dimethacrylate) (Net-IA); photos of prototypes manufactured by film preparation. Dimensions of probes are according to ASTM D-638 type 5, (**e**) poly(lactic acid) (PLA)/IA blend; (**f**) PLA/PIA blend; (**g**) PLA/Cop-IA blend; (**h**) PLA/Net-IA blend.

The PLA blends with itaconic acid (IA), poly(itaconic acid) (PIA), poly(itaconic acid)-*co*-poly(methyl itaconate) (Cop-IA), and *net-*poly(itaconic acid)-*ν*-triethylene glycol dimethacrylate (Net-IA) were made at a temperature of 170 °C due to the melting temperature of IA, discussed in the section on thermogravimetric analysis. Scheme 1 illustrates the synthesis paths of the different polymeric architectures. PIA was synthesized with excess potassium persulfate at a temperature of 50 °C and a polymerization time of 72 h. It is well known that IA polymerization generates complex structures according to the type of initiator [35], controlled radical polymerization [36], synthesis conditions [35], and radical polymerization (transfer chain reactions) [37]. Therefore, the intrinsic properties of the synthesized polymer are difficult to compare.

Moreover, the random copolymer was synthesized from the esterification of PIA with methanol using *p*-toluenesulfonic acid as a catalyst. From a purification point of view, these synthesis pathways were less challenging than the esterification of IA with methanol. On the other hand, IA was also crosslinked with TEGMA and KPS initiators at a temperature of 60 °C. The obtaining of microspheres was evident from the moment of polymerization.

### 3.1. Fourier Transform Infrared Spectroscopy

**Figure 1:**
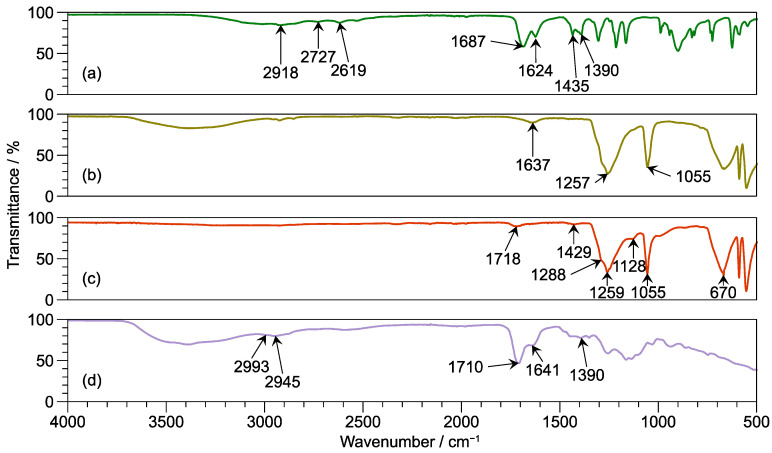
FTIR spectra. (**a**) IA; (**b**) PIA; (**c**) Cop-IA; (**d**) Net-IA.

Infrared spectroscopy was employed before mixing PLA with IA or its architectures to understand their differences. The FTIR spectrum of IA exhibits a broad band at 3385–2400 cm^−1^, which is attributed to the O–H stretching vibration as a result of the presence of hydrogen bonding; see Figure 1a. In addition, the peak corresponding to the stretching vibration of the carbonyl group is shown at 1687 cm^−1^ owing to intramolecularly hydrogen-bonded acids (as a dimer). For the CH_2_–CO group, the methylene group presents a deformation vibration at 1435 cm^−1^. These signals are characteristic of carboxylic acid groups. At 1624 cm^−1^, the C=C stretching vibration is displayed. The peaks corresponding at 1435 (asymmetric deformation) and 1390 cm^−1^ (symmetric band) are assigned to the methyl group adjacent to the carbonyl groups [38]. Likewise, the band at 2918 cm^−1^ is assigned to the symmetrical vibration of methylene (CH2) [38].

Figure 1b displays the spectrum of the PIA homopolymer. The broad band at 3800–2750 cm^−1^ is attributed to the O–H stretching vibration. In addition, the peaks at 2922 cm^−1^ and 2856 cm^−1^ are assigned to the asymmetric and symmetrical C–H stretching vibrations, respectively. Finally, the peaks at 1637, 1257, 663, 588, and 551 cm^−1^ are attributed to the dimer’s presence of the COOH groups [39].

The spectrum of Cop-IA is shown in Figure 1c. The peaks at 2924 cm^−1^ and 2854 cm^−1^ are attributed to the asymmetrical and symmetrical stretching vibrations of the C–H bond. The peak at 1718 cm^−1^ is assigned to the carbonyl group stretching vibration of the ester group. The band at 1429 cm^−1^ corresponds to the asymmetrical CH_3_ deformation vibration. Similarly, the peak at 1128 cm^−1^ (rocking vibration) is attributed to the methyl ester group. These signals confirm the formation of the copolymer from the esterification of the homopolymer with methanol [8]. Similar to PIA, the non-esterified COOH groups associate in the form of dimers, according to the observed bands at 1259, 670, 590, and 551 cm^−1^ corresponding to the stretching vibration of C–O [39,40]. The methyl content was determined based on deconvolution from the peak to 1718, with a value of 35%; see Figure S1 [11,41].

Figure 1d illustrates the FTIR spectrum of Net-IA. The band corresponds to the O–H stretching vibration, which is located between 3385 cm^−1^ and 2500 cm^−1^. The peaks at 2993 and 2945 cm^−1^ correspond to the stretching vibration of the C–H bond. At 1710 cm^−1^, the stretching vibration of the carbonyl (C=O) of the ester group, particularly the crosslinking agent, is observed [42]. The peak of 1641 cm^−1^ is assigned to the stretching vibration of the carbonyl group of IA. On the other hand, the peaks at 1350 cm^−1^ and 1390 cm^−1^ are attributed to the combination of C–O bond vibrations and O–H deformation.

**Figure 2:**
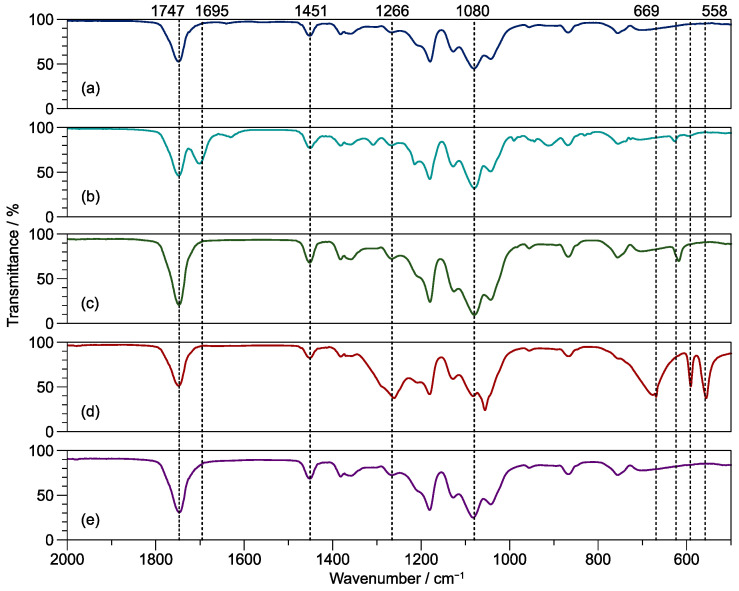
FTIR spectroscopy of PLA/itaconic acid architectures at 10 wt.% compared to PLA. (**a**) (PLA); (**b**) PLA/IA10; (**c**) PLA/PIA10; (**d**) PLA/Cop-IA10; (**e**) PLA/Net-IA10.

On the other hand, the blend’s constituents have similar groups; for this reason, the mixtures were analyzed at a 10% content of itaconic acid and its architectures. Figure 2 illustrates the infrared spectra of PLA/IA and its architecture blends and their comparison with PLA. The PLA/IA10 blend shows the characteristic band corresponding to the stretching vibration of the CO at 1695 cm^−1^. Likewise, the C=C stretching vibration is observed at 1624 cm^−1^. This molecular vibration is essential because it suggests that a process of self-polymerization does not occur during the formation of the PLA/IA10 blend at a temperature of 170 °C for 2 min.

The spectra of the PLA/PIA10 and PLA/Cop-IA10 blends show significant differences in some of these bands at 1266, 669, 620 590, and 558 cm^−1^, which are attributed to dicarboxylic acid due to the in-plane vibration of the O–CO group, i.e., dimers. However, the band at 1266 cm^−1^ in PLA/Cop-IA10 is more intense because a methyl ester stretching vibration also occurs. Despite the absence of these bands in the PLA/Net-IA10 spectrum, it suggests that PLA covers the Net-IA microspheres. Thus, the PLA/Net-IA10 spectrum is similar to the PLA spectrum.

In conclusion, based on the results, the chemical structures of IA, PIA, and Cop-IA showed a hydrogen bond in the form of a dimer between two carboxylic acids of a different IA, PIA, or Cop-IA, i.e., a head-to-tail bond. The polymerization of IA was successful due to the absence of the band corresponding to the alkene group. Likewise, the crosslinking between IA and TEGMA displayed substantial changes compared to its precursors. The esterification of PIA with methanol proved to be an easier method for obtaining copolymers because it eliminates the monomer purification process. Compared to IA and its architectures, the PLA/IA10, PLA/PIA10, and PLA/Cop-IA10 blends also exhibit the formation of dimers.

### 3.2. Electron Microscopy

**Figure 3:**
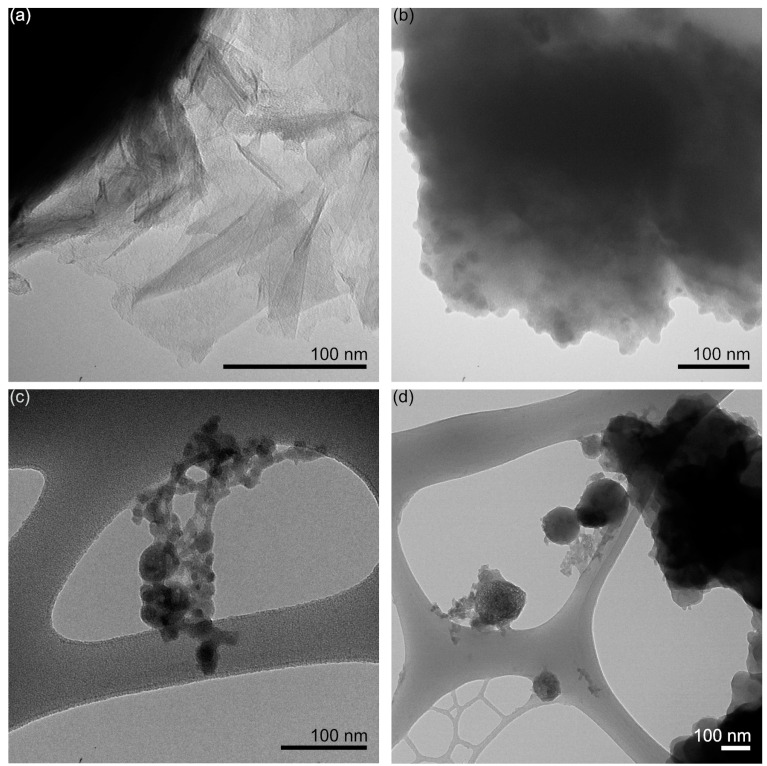
TEM micrographs. (**a**) IA; (**b**) PIA; (**c**) Cop-IA; (**d**) Net-IA.

The morphology analysis by transmission electron microscopy (TEM) of IA and its architectures was conducted in order to elucidate the differences presented by these materials. Figure 3 illustrates the TEM micrographs of (a) IA, (b) PIA, (c) Cop-IA, and (d) Net-IA. The micrograph of IA shows thin layers similar to graphene. Harlow R.L. and Pfluger C.E. reported that the crystal structure of IA (orthorhombic, *Pbca*) is composed of molecules that are hydrogen-bonded in a head-to-tail fashion to form infinite chains in a direction (a-axis) [43]. Arun Renganathan et al. reported that IA exhibited several intra- and intermolecular hydrogen bond interactions [44]. They also theoretically studied the possibility that IA can present molecular packing along the a-, b-, and c-axes [44]. Therefore, the observed morphology of IA corresponds to the inter- and intramolecular interaction of hydrogen bonds forming a packing in one direction. This result coincides with the infrared spectroscopy, where the formation of dimers is observed.

The TEM micrographs of the IA architectures, in general, exhibit a spherical morphology; see Figure 3b–d. IA, PIA, Cop-IA, and Net-IA have a particle size of 20–30, 80–250, and 200–600 nm, respectively. Figure 3b shows the TEM micrograph of PIA, and the formation of clusters of the homopolymer is observed due to the interaction between the polymer chains and the functional groups present in them. The sample preparation methodology, which utilized isopropyl alcohol, explains this result. As PIA is not soluble and has more groups of carboxylic acid, it undergoes an agglomeration and is observed with spherical particles at the edges.

Figure 3c presents the TEM micrograph of Cop-IA, showing an amorphous structure with areas with a higher electron density, where copolymer aggregates are located. Finally, Figure 3d displays the TEM micrograph of Net-IA, where the surface of the sphere has a rough texture, suggesting the crosslinking of the polymeric structures. Likewise, small nodules are observed on the surface due to the polymerization of IA and TEGDMA, together with the formation of the three-dimensional network in the material. Similarly, Alrahlah et al. report that spherical conformation particles of regular size and micro holes are observed on the surface [45].

**Figure 4:**
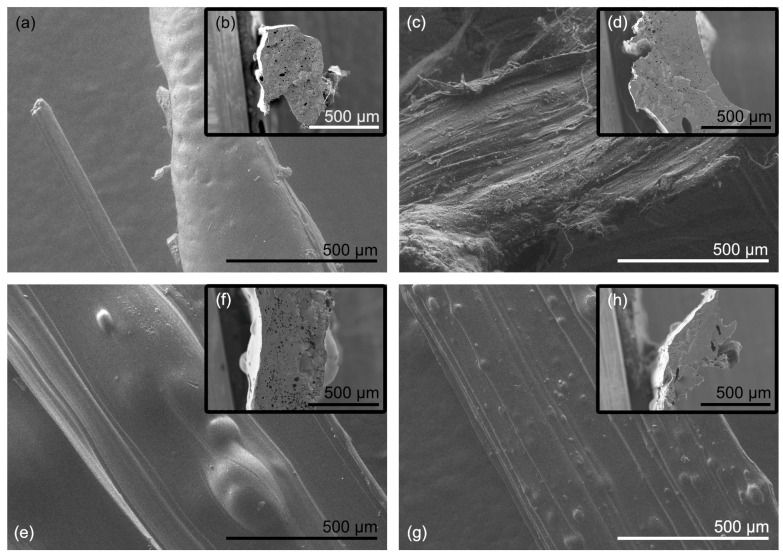
SEM micrographs of PLA/itaconic acid architectures at 10% wt. (**a**) Surface PLA/IA10; (**b**) cross-section PLA/IA10; (**c**) surface PLA/PIA10; (**d**) cross-section PLA/PIA10; (**e**) surface PLA/Cop-IA10; (**f**) cross-section PLA/Cop-IA10; (**g**) surface PLA/Net-IA10; (**h**) cross-section PLA/Net-IA10.

The morphology of the PLA/IA10, PLA/PIA10, PLA/Cop-IA10, and PLA/Net-IA10 blends was evaluated through SEM. Figure 4a illustrates the SEM micrograph of the PLA/IA10 blend, showing fibers and roughness formed on the surface. At low concentrations of IA, i.e., 0.1 wt%, Figure S2, and 1 wt%, Figure S3, the morphology of the PLA/IA blend does not suffer significant alterations. Meanwhile, when the concentration increases to 3%, Figure S4 is identical to PLA/IA10. The PLA/IA blend morphology can be attributed to chemical or physical interactions between IA and PLA, where the additive forms nucleation points in the polymer matrix, which promotes the alignment of the polymer in the form of fibers.

The SEM micrographs of the PLA/PIA10 blends are illustrated in Figure 4c. These blends exhibit a behavior similar to that described above in the PLA/IA blends, where at low concentrations, e.g., 0.1 wt%, Figure S5, and 1 wt%, Figure S6, PIA does not have a significant incidence on the morphology of PLA. The interaction between PLA and PIA is visible at 3 wt%, Figure S7, and 10 wt%. For the PLA/PIA blend, it is suggested that the homopolymer generates nucleation points that promote the formation of fibrillar structures.

Figure 4e displays the micrograph of the PLA/Cop-IA10 blends obtained by SEM. The copolymer particles embedded in the polymer matrix are observed in various concentrations. In the same way, Cop-IA has acceptable compatibility with the polymer, promoting the formation of fibrillar structures, where the surface is maintained with a smooth texture.

The SEM micrograph of the PLA/Net-IA10 blends is shown in Figure 4g. The microspheres are embedded and evenly distributed in the polymer matrix. In turn, PLA fibers with a smooth texture are formed. This result can be explained due to the physical interactions between Net-IA and the polymeric material. Nevertheless, these interactions were not detected by infrared spectroscopy because the interface between the Net-IA microsphere and PLA has to be analyzed. On the other hand, Net-IA and Cop-IA have similar behavior in the PLA matrix.

Figure 4b,d,f,h illustrate the cross-section of the PLA/IA10, PLA/PIA10, PLA/Cop-IA10, and PLA/Net-IA10 blends, respectively. All blends, except PLA/Net-IA10, showed a porous microstructure that formed throughout the fibrillar material.

In summary, it is clear that the microstructures obtained after the melting blend process are of the fibrous type, which could be applied to develop fabrics or the 3D printing of filament.

### 3.3. Rheology

**Figure 5:**
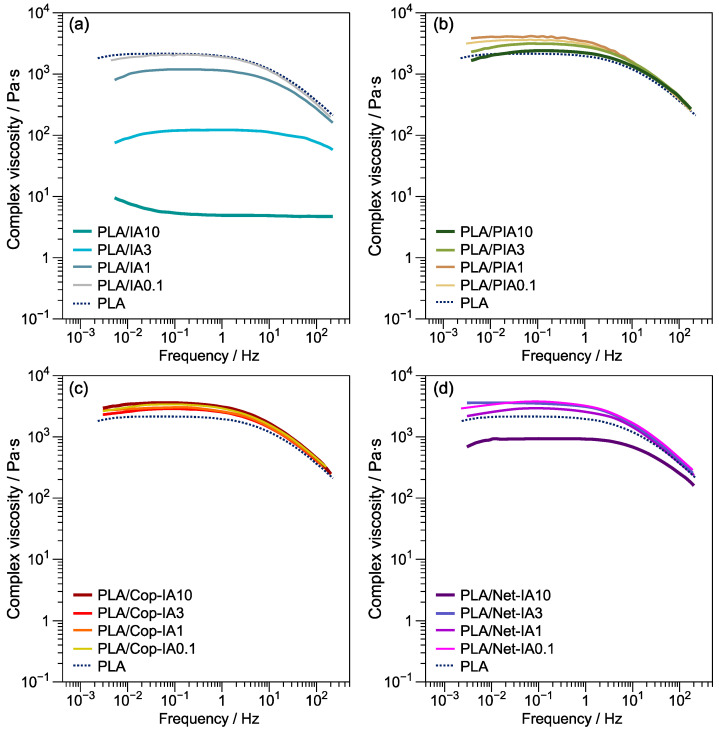
Complex viscosity (η*) of PLA and (**a**) PLA/IA; (**b**) PLA/PIA; (**c**) PLA/Cop-IA; (**d**) PLA/Net-IA. All materials were measured at 170 °C.

Rheological measurements were performed to understand and to obtain information, including the miscibility, phase behavior, structure, and shear thinning behavior, among others, of the blends. Figure 5 displays that the complex viscosity (η*) values for PLA/PIA, PLA/Cop-IA, and PLA/Net-IA are similar or superior to those of neat PLA at all frequencies, except for PLA/Net-IA10, which is lower and more identical to the PLA/IA1 blend. In contrast, the PLA/IA blend values have a singular rheological behavior and are completely different at all frequencies, whereas the PLA/IA10 blend exhibits a minor complex viscosity compared to all blends. It is important to note that a high concentration of IA is extremely plasticizing.

On the contrary, the blends containing 1, 3, and 10 wt% of IA exhibit a Newtonian plateau at low frequencies. In addition, it is evident that IA acts as a plasticizer by decreasing the η*. Benkraled et al. demonstrated the behavior of a plasticizer on η* when PLA was mixed with PEG using the Carreau–Yasuda model [9]. Ivorra-Martinez et al. observed that the DBI content in the PLA matrix results in a decrease in viscosity owing to a reduction in the intermolecular interactions in the polymer chains, creating a plasticizing effect [46].

Table 1 illustrates a comparison of the η* obtained at a frequency of 0.1 Hz and the Carreau–Yasuda model. The results of the Carreau–Yasuda model were calculated using Equation (S1), and Table S1 displays the parameters used for the model. Although there is a difference between the two criteria, the results obtained at 0.1 Hz are comparable to the Carreau–Yasuda model. The IA present in the PLA/IA blends has a plasticizing effect, and it is observed that it drastically decreases the η* when the mixture contains 10 wt% of IA. Low concentrations of PIA, Cop-IA, and Net-IA in the PLA/PIA, PLA/Cop-IA, and PLA/Net-IA blends can be utilized to reinforce, as revealed by the findings. However, the PLA/PIA10 blend has a η* similar to PLA, so it is necessary to study high concentrations of PIA to determine whether it has a plasticizing effect. On the contrary, the PLA/Net-IA10 blend significantly decreases, suggesting a plasticizing effect at this concentration.

**Figure 6:**
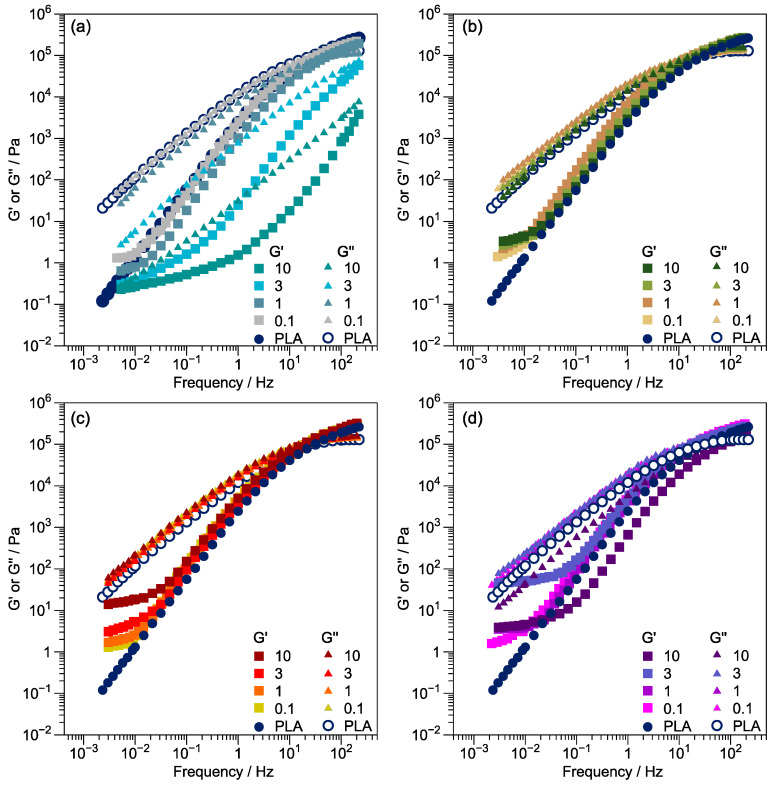
Master curves of storage modulus (G’, square) and loss modulus (G”, triangles) for (**a**) PLA/IA; (**b**) PLA/PIA; (**c**) PLA/Cop-IA; (**d**) PLA/Net-IA; using data at 160, 170, and 200 °C.

Figure 6 displays the plots of storage modulus (G’) and loss modulus (G”) versus frequency for the PLA/IA, PLA/PIA, PLA/Cop-IA, and PLA/Net-IA blends. For PLA/IA, Figure 6a, the G’ decrease concerning IA concentration is due to a decrease in molecular entanglements. Likewise, the behavior of the G” is typical of a plasticizing effect [9]. In contrast, the G’ of the PLA/PIA, PLA/Cop-IA, and PLA/Net-IA blends is equivalent to or higher than neat PLA, except for the PLA/Net-IA10 blend, which is similar to the PLA/IA blend. The increase in the G’ of the blends compared to PLA is attributed to an increased chain entanglement [25].

Huerta-Angeles et al. reported that the enhancement in the molecular weight of PIA leads to an enhancement in the viscosity of the aerogel. They also found that the G’ and G” of the PIA/laponite hydrogel increased with a higher frequency, which can be attributed to the enhanced hydrogen bonds [47]. In the same way, Kwon et al. reported that the addition of IATG (IA/1-thioglycerol) to polyurethane results in an increase in the storage modulus. They attributed this behavior to the carboxyl groups, which generate an increase in physical crosslinking in the polyurethane chains by forming more hydrogen bonds [48].

Finally, the compatibility between PLA and IA and its architecture can be elucidated through the correlations of G’ vs. G”, known as Han plots; see Figure S8. The Han plots display linear behavior for the PLA/IA blend, indicating a homogeneous structure, and the blend demonstrates excellent compatibility, according to the results of Yang et al. [49]. For the PLA/PIA, PLA/Cop-IA, and PLA/Net-IA blends, a nonlinear correlation is observed with an increasing additive concentration, indicating immiscibility in the blends [50,51].

In summary, the η*, G’, and G” of the PLA/IA blend suggest a behavior as a plasticizer; on the contrary, the rest of the architectures have a reinforcing effect on PLA.

### 3.4. Differential Scanning Calorimetry

The glass transition temperature (T_g_) and melting temperature (T_m_) for all blends were obtained from the second heating by DSC; see Table 2. The DSC thermograms of the PLA/IA, PLA/PIA, PLA/Cop-IA, and PLA/Net-IA blends (Figures S9–S12) show a decrease in the T_g_ compared with neat PLA, especially for PLA/IA10 and PLA/Cop-IA10, which exhibit a decrease of 23% and 11%, respectively. This behavior could be explained by the fact that IA and its architectures act as plasticizers, providing greater mobility in the amorphous domains of the polymeric matrix and favoring ductility.

Additionally, Ivorra-Martinez et al. reported a decrease in the T_g_ for mixtures of diester itaconates with PLA [46]. Spasojevic et al. reported a decreased T_g_ when incorporating dibutyl itaconate into poly(methyl methacrylate). The flexible ester groups of the itaconate provide a plasticizing effect [52]. Zhao et al. observed a decrease in the T_g_ of the PLA/citric acid/PEG blend at low concentrations and those superior to 15%, attributed to a reduction in the intermolecular forces in the polymer matrix, producing a plasticizing effect. However, when 15% PEG/CA was incorporated, there was an increased T_g_ of the blend owing to the enhanced branched PEG/CA, forming copolyesters with the PLA matrix, generating greater entanglement between the chains of the blend, and limiting the movement of the polymer chains [25].

On the other hand, the PLA/IA, PLA/PIA, PLA/Cop-IA, and PLA/Net-IA blends show a decrease in the T_m_. Compared with PLA (T_m_ = 153.6 °C), the PLA/IA10, PLA/PIA10, PLA/Cop-IA10, and PLA/Net-IA10 blends have a value close to 150 °C, highlighting the incorporation of IA at 10 wt.%, where a T_m_ of 147.9 °C is reached.

All blends’ cold crystallization temperature (T_cc_) is close to 120 °C. Marsilla et al. observed that the T_cc_ decreases with higher IA content due to the steric effect of the additive that prevents crystal growth [53]. Ivorra-Martinez et al. reported that increasing the DBI content enhances the T_cc_ of the PLA/DBI blend [46].

The mixtures exhibit different behaviors in the enthalpy of crystallization (ΔH_cc_). For instance, the PLA/IA blend increases the ΔH_cc_ concerning the concentration of IA. Nevertheless, significant ΔH_cc_ changes were not observed in the PLA/PIA, PLA/Cop-IA, or PLA/Net-IA blends at all concentrations. Finally, similar behavior is observed in the fusion enthalpy of polymer blends.

The crystallinity degree (χ_cc_) was determined using Equation (S2) for all blends; see Table 2. Due to the proximity between the crystallization and enthalpy peaks of the second heating cycle, the first derivative was used to establish the limits of the integral for determining the enthalpy; see Figure S13. The supplementary material contains the DSC thermograms and the χ_cc_ equation; see Figures S9–S12. Compared with PLA, the PLA/IA blend exhibits the maximum χ_cc_ when it contains 3 wt% of IA. Nevertheless, the PLA/PIA blends decrease their χ_cc_ relative to the increase in PIA concentration. In contrast, the PLA/Cop-IA and PLA/Net-IA blends enhance their χ_cc_ depending on the Cop-IA or Net-IA concentration. These results are consistent with those reported by Zhao et al. [25]. Similarly, Hu et al. observed an increase in the χ_cc_ with the addition of citric acid to the polymer matrix, as it restricts the chain mobility [54].

The variations in the χ_cc_ observed in the DSC were validated by the XRD. It is well known that the XRD spectrum of neat PLA exhibits two characteristic peaks at angle 2θ = 16.6° and 19.3°, corresponding to the crystalline structure of PLA [55]. Furthermore, for these peaks to be observed under this characterization technique, it is necessary to perform the analysis at a temperature of 80 °C or a heat treatment [55].

**Figure 7:**
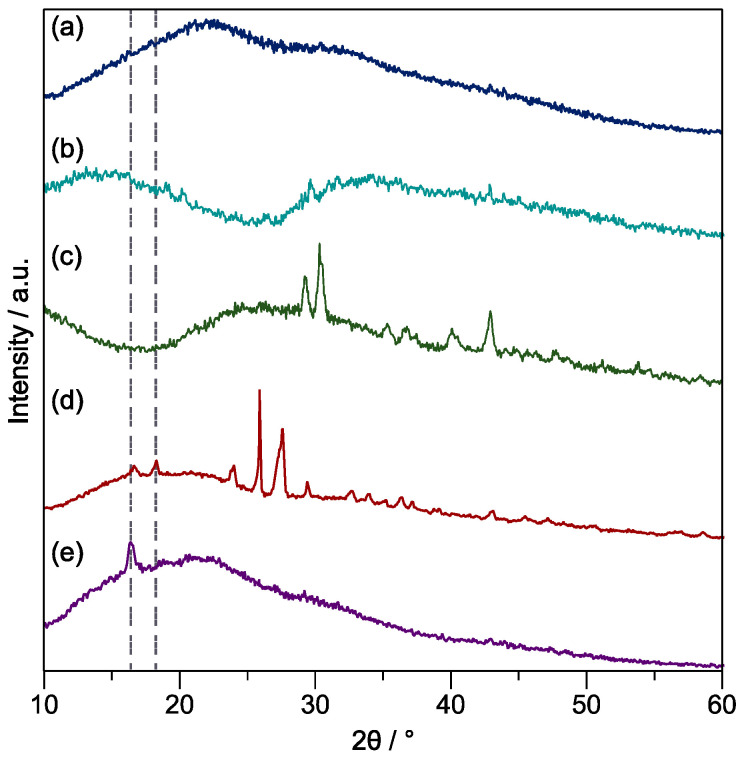
X-ray diffraction. (**a**) PLA; (**b**) PLA/IA10; (**c**) PLA/PIA10; (**d**) PLA/Cop-IA10; (**e**) PLA/Net-IA10.

Figure 7 displays the XRD analysis of the PLA/IA10, PLA/PIA10, PLA/Cop-IA10, and PLA/Net-IA10 blends and PLA. Compared to PLA, the PLA/IA10 and PLA/PIA10 blends do not show the characteristic peaks of crystallinity of PLA, and even the PLA/PIA10 blend is more amorphous than the PLA/IA blend. Nevertheless, these peaks can be observed only in the PLA/IA3 and PLA/PIA0.1 blends; see Figures S14 and S15, respectively. Therefore, this behavior is consistent with the DSC findings. In contrast, the PLA/Cop-IA and PLA/Net-IA blends show this pair of peaks. Low concentrations are also observed to highlight the PLA/Net-IA blend because all Net-IA concentrations are shown; see Figures S16 and S17. This evidence suggests that the Cop-IA and Net-IA architectures restrict the mobility of the PLA chains, and hence, the particles generate nucleation points. However, the Net-IA architecture more substantially affects the χ_cc_ than the Cop-IA does due to the particle size. Sa’adah et al. observed an increase in the crystallinity peak of the PLA/PVA/Starch blend with the addition of citric acid [56].

On the other hand, the PLA/PIA10 and PLA/Cop-IA10 blends display the characteristic peaks of PLA, as well as additional peaks reported for the structure based on itaconic acid [57].

### 3.5. Dynamic Mechanical Thermal Analysis

**Figure 8:**
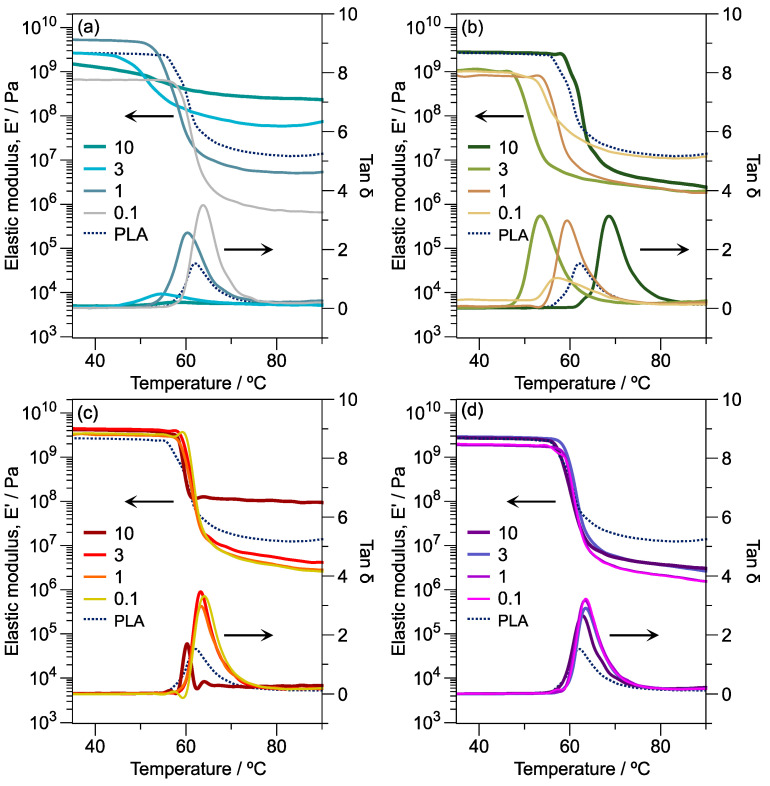
Dynamic mechanical thermal curves. (**a**) PLA/IA; (**b**) PLA/PIA; (**c**) PLA/Cop-IA; (**d**) PLA/Net-IA.

Dynamic mechanical thermal analysis (DMTA) is a polymer characterization technique with far more sensitivity to macroscopic and molecular relaxation processes than thermal analysis techniques. Figure 8 displays the dynamic mechanical relaxation spectra for the PLA/IA, PLA/PIA, PLA/Cop-IA, and PLA/Net-IA blends. The T_g_ determination under this technique is that it is a direct method and was calculated with the Tan δ maximum, see Table S2. The Tg’s behavior is altogether unique compared to the DSC. The PLA/IA blends display a decrease in the T_g_, with the minimum in the PLA/IA3 (54.1 °C) blend. The PLA/PIA, PLA/Cop-IA, and PLA/Net-IA blends have a T_g_ close to that of PLA (61.4 °C). The PLA/Cop-IA0.1 and PLA/Net-IA3 blends displayed the highest values at 64.4 and 64.2 °C, respectively. Therefore, the incorporation of IA in PLA has a plasticizing effect, and PIA, Cop-IA, and Net-IA have no significant changes on the T_g_ of PLA.

Figure 8 displays the DMTA curves in the region’s glassy state, glass–rubber relaxation, and rubbery plateau of PLA. Compared with the elastic modulus (*E*’) of PLA (2733 MPa), the PLA/IA1, PLA/PIA10, PLA/Cop-IA0.1, PLA/Cop-IA1, PLA/Cop-IA3, PLA/Cop-IA10, PLA/Net-IA3, and PLA/Net-IA10 blends have higher *E*’ values, which are 5284, 2769, 3463, 3272, 4421, 4200, 2889, and 2736 MPa, respectively. The *E*’ was determined at a temperature of 35 °C. Liu et al. found that IA produced an increase in the *E*’, which was attributed to the intermolecular repulsion between the polyacrylate chains and IA [58]. The PLA/Cop-IA blends are highlighted, since they all outperformed PLA. This result may be explained by the fact that the additive used in the PLA blend needs a balance of hydrophilic and hydrophobic groups to have an adequate miscibility and, therefore, an increase in the *E*’ [59].

On the other hand, the tan δ for the PLA/IA blend diminishes as the IA content increases, suggesting a plasticizing effect; see Figure 5a. Ivorra-Martinez et al. reported a decrease in the tan δ, indicating a high level of plasticization in the PLA-DBI blends, resulting in a ductile material at room temperature [30,46]. The tan δ of PLA/PIA0.1 and PLA/Cop-IA10 had a behavior similar to neat PLA; see Figure 8b,c. The rest of the mixtures have a tan δ above that of neat PLA. Spasojevic et al. observed an increase in the tan δ concerning the itaconate content in the PMMA matrix. They attributed this effect to the interaction of the aliphatic chains of the itaconates with the polymer [52].

### 3.6. Thermogravimetric Analysis

**Figure 9:**
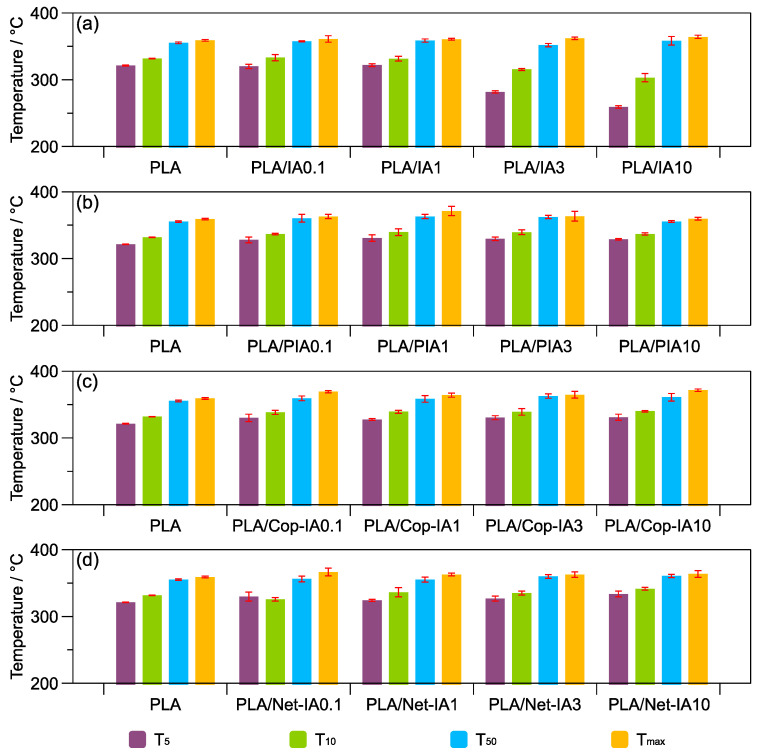
The thermal stability at a weight loss of 5% (T_5_), 10% (T_10_), and 50% (T_50_) and at the maximum decomposition temperature (T_max_), according to the first derivative of the TGA curve. (**a**) PLA/IA0.1, PLA/IA1, PLA/IA3, PLA/IA10, and PLA. (**b**) PLA/PIA0.1, PLA/PIA1, PLA/PIA3, PLA/PIA10, and PLA. (**c**) PLA/Cop-IA0.1, PLA/Cop-IA1, PLA/Cop-IA3, PLA/Cop-IA10, and PLA. (**d**) PLA/Net-IA0.1, PLA/Net-IA1, PLA/Net-IA3, PLA/Net-IA10, and PLA.

The evaluation of the thermal stability of the PLA/IA, PLA/PIA, PLA/Cop-IA, and PLA/Net-IA blends is necessary due to the decomposition of the chains that make up these additives. For this reason, the temperature was evaluated at a weight loss of 5% (T_5_), 10% (T_10_), 50% (T_50_), and at the maximum decomposition temperature (T_max_) according to the first derivative of the TGA curve; see Figure 9. Table S3 illustrates the thermogravimetric results for the PLA/IA, PLA/PIA, PLA/Cop-IA, and PLA/Net-IA blends at T_5_, T_10_, T_50_, and T_max_.

At a concentration of 1%, the PLA/IA and PLA/PIA blends experience the highest T_5_, reaching 321.9 °C and 330.8 °C, respectively. Meanwhile, thermal stability is achieved at a concentration of 10%, i.e., T_10_, for the PLA/Cop-IA and PLA/Net-IA blends. Compared to neat PLA, the thermal stability is favored, suggesting that Cop-IA and Net-IA produce a higher ordering in the polymer chains, as observed in the SEM images, where the fibrillar structures of the polymer are obtained. On the other hand, as the concentration of the IA in the blend increases, there is a decrease in the thermal stability owing to the interactions between the polymer matrix and the monomer. This result is verified by the DSC and DMTA analyses, in which a considerable decrease in the T_g_ is observed.

Neat PLA has a T_max_ of 359.1 °C, but it varies in the PLA/IA, PLA/PIA, PLA/Cop-IA, and PLA/Net-IA blends. For example, the T_max_ for PLA/IA1, PLA/PIA1, PLA/Cop-IA1, and PLA/Net-IA1 is 360.6 °C, 371.3 °C, 364.1 °C, and 362.7 °C, respectively. Therefore, these architectures promote thermal stability due to their interactions at the structural level.

A recent study by Liu et al. identified that the addition of IA to polyacrylate increases the thermal stability due to the stearic hindrance of IA and the repulsive forces that occur in the polyacrylate chains, forming compact structures with a higher T_g_ [58]. In an analysis of the incorporation of citric acid into starch, Chen et al. found that it generates strong intermolecular interactions (hydrogen bonds); however, at high concentrations of citric acid, the thermal stability decreases, attributed to acid hydrolysis that reduces the degree of polymer crosslinking [19]. Likewise, Wang et al. demonstrated that the T_5%_ decreases with the increase in citric acid content for thermoplastic starch [60].

On the other hand, the thermogravimetric trace of IA is given in Figure S17. The sample displays a single mass loss over a fairly broad temperature range. Until 150 °C, the sample is stable, but it rapidly loses 98% of its mass at 221 °C. In this stage, two endothermic phenomena occur, according to the DSC. Except for IA, the values obtained from T_m_ are from the first heating. The first is the melting point at 176 °C (160–200 °C), and the second is the decomposition of IA from 200 to 260 °C. The mass loss during the melting point is close to 11% up to 200 °C. The processing conditions of the PLA/IA blends and their different architectures are at 170 °C, with a mass loss of about 1%. In contrast, the mass loss of the second endothermic peak is close to 86% due to the decarboxylation and dehydration of IA. Silva et al. reported the TGA and DTG thermograms for citric acid, in which the decomposition occurs in one step (150–220 °C) due to the dehydration and decarboxylation processes [61].

### 3.7. Mechanical Analysis by DMA

Table 3 illustrates the DMA results for the PLA/IA, PLA/PIA, PLA/Cop-IA, and PLA/Net-IA blends. The incorporation of IA has a plasticizing effect on the PLA matrix. Compared with neat PLA, the PLA/IA0.1 and PLA/IA1 blends exhibit an increase in the elongation at break of 1220% and 106%, respectively. Similarly, the E’ and σ increased by 93% and 27%, respectively, with the concentration of PLA/IA1; however, as the IA content increases, the E’ and ε decrease. Ivorra-Martinez observed this effect in PLA-DBI blends, where this additive increases the free volume between the polymer matrix chains [46]. The plasticizing effect is also observed with organic acids, e.g., citric acid, which reduces the polymer chain interactions, replacing them with hydrogen bonds between the additive and the bioplastic, resulting in increased flexibility on the polymer [56].

Moreover, PLA/IA10 shows a reduction of 41% in the ε. Krishnan et al. described this effect in polymer blends, attributing it to phase separation or material agglomeration due to the high additive content [29].

The PLA/PIA0.1 and PLA/PIA1 blends exhibit a plasticizing effect, achieving an increase of 417% and 517% in the ε, respectively. On the other hand, the PLA/PIA3 blend decreases by 6% and 39% for the ε and E’, respectively. In contrast, the PLA/Cop-IA3 and PLA/Cop-IA10 blends show a 69% increase of 62% and 53% in the E’, respectively. This result is consistent with Ivorra-Martinez’s findings, where IA esters are a reliable option for bioplastic plasticization [30].

The PLA/Net-IA3 blend exhibits a 5% increase in the E’ and a 32% decrease in the σ compared to neat PLA. This behavior was reported by Ivorra-Martinez et al., attributing it to the crosslinking in the material’s chemical structure, restricting the mobility of the polymer chains [30]. Likewise, Spasojevic et al. reported that the incorporation of dimethyl itaconate (DMI) into PMMA creates micro-defects in the polymer chains, causing a decrease in the tensile strength and elongation at break [52]. In the same way, Lazouzi et al. reported PMMA/DMI (95:5 wt%) blends where the addition of DMI to the polymer increased the elastic modulus and decreased the elongation at break, attributed to the incorporation of pendant groups by DMI, which creates micro-defects in the polymer structure [62]. In another work, Liu et al. reported that the elastic modulus increased and the elongation at break decreased with the IA content, attributed to the repulsive forces between the polyacrylate chains and IA [58]. Therefore, the PLA/IA and PLA/PIA blends show plasticization effects at low concentrations, in particular 0.1 and 1.0 wt%, owing to the physical interactions between IA or PIA and PLA.

## 4. Conclusions

In the present investigation, the diverse chemical architectures from itaconic acid (IA), including poly(itaconic acid) (PIA), poly(itaconic acid)-*co*-poly(methyl itaconate) (Cop-IA), and *net*-poly(itaconic acid)-*ν*-triethylene glycol dimethacrylate (Net-IA), were synthesized. The carboxylic acids present in these chemical structures formed dimers, i.e., interactions of the hydrogen bonds between two different units of IA, PIA, or Cop-IA.

Moreover, poly(lactic acid) (PLA)/IA, PLA/PIA, PLA/Cop-IA, and PLA/Net-IA blends were developed and studied using various characterization techniques. All blends were prepared with 0.1, 1, 3, and 10 wt% of IA, PIA, Cop-IA, or Net-IA. The formation of the dimers between the carboxylic acids was confirmed by infrared spectroscopy for the PLA/IA, PLA/PIA, and PLA/Cop-IA blends. Because of this, fibrillar structures for the PLA/IA, PLA/PIA, PLA/Cop-IA, and PLA/Net-IA blends were observed. Additionally, the cross-sectional evaluation of the fibers revealed the presence of porous microstructures throughout the material, except for the PLA/Net-IA blend.

The incorporation of IA into a polymeric blend can be carried out at 170 °C via melting blend, without the processes of decarboxylation and self-polymerization. However, IA is recommended to be used at a concentration of less than 1 wt%; at high concentrations, it is an extreme plasticizer, according to the complex viscosity, storage modulus, and loss modulus of the PLA/IA blend. On the contrary, PLA/PIA, PLA/Cop-IA, and PLA/Net-IA have a reinforcing effect on the PLA.

The PLA T_g_ decreases in the PLA/IA blend, while the values for the other blends are similar. The tan δ for the PLA/IA blend also diminishes as the IA content increases, suggesting a plasticizing effect. The *E*’s (at 35 °C) of the PLA/Cop-IA blends are highlights, since they all outperformed PLA. In the rubbery plateau, the *E*’ for the PLA/IA3, PLA/IA10, and PLA/Cop-IA10 blends is superior to that of PLA. These blends are stiffer than PLA, e.g., 80 °C, so they could be applied as disposable food packing.

The crystallinity degree displays higher values for blends that contain Net-IA > Cop-IA than IA > PIA. Net-IA enhances the crystallinity degree because it restricts the mobility of PLA. Likewise, the PLA/Net-IA3 blend exhibits a 5% increase in the E’ and a 32% decrease in the σ compared to neat PLA. On the other hand, the PIA, Cop-IA, and Net-IA architectures promote thermal stability in the PLA blends.

Finally, the properties of PLA are influenced by IA and its diverse chemical architectures, especially PIA, Cop-IA, and Net-IA. The potential applications may be several, such as disposable food packaging, fabrics, medical surgery products, and additive manufacturing, among others.

## Figures and Tables

**Table 1:** The comparison of the complex viscosity (η*) at 0.1 Hz and the Carreau–Yasuda model.

Sample	η* at 0.1 Hz (Pa·s)	η_0_, Carreau–Yasuda Model (Pa·s)
PLA	2140	2073.1
PLA/IA0.1	2038.4	1999.3
PLA/IA1	1189.0	1175.7
PLA/IA3	118.7	113.8
PLA/IA10	5.35	---
PLA/PIA0.1	3579.3	3568.9
PLA/PIA1	4059.0	4118.4
PLA/PIA3	3139.2	3020.1
PLA/PIA10	2400.5	2252.5
PLA/Cop-IA0.1	3453.4	3471.1
PLA/Cop-IA1	3066.9	3010.0
PLA/Cop-IA3	2877.4	2806.1
PLA/Cop-IA10	3594.7	3584.1
PLA/Net-IA0.1	3553.7	3607.7
PLA/Net-IA1	2914.2	2897.8
PLA/Net-IA3	3455.2	3545.9
PLA/Net-IA10	910.6	858.71

**Table 2:** Values were obtained from DSC curves of PLA/IA, PLA/PIA, PLA/Cop-IA, and PLA/Net-IA blends vs. PLA.

Sample	T_g_ ^1^ (°C)	T_cc_ ^2^	ΔH_cc_ ^3^	T_m_ ^4^ (°C)	ΔH_m_ ^5^	χ_cc_ ^6^ (%)
PLA	65.2	122.9	8.6	153.6	19.8	12
PLA/IA0.1	56.5	116.4	7.5	149.4	16.3	9.5
PLA/IA1	58.5	119.5	11.3	148.5	19.3	8.7
PLA/IA3	49.0	121.3	11.5	147.9	20.9	11.2
PLA/IA10	51.2	114.6	13.7	146.5	19.5	6.4
PLA/PIA0.1	59.6	122.0	4.0	152.2	16.3	13.2
PLA/PIA1	59.3	124.1	8.9	149.9	15.1	6.9
PLA/PIA3	59.1	123.7	10.1	150.4	15.8	6.2
PLA/PIA10	59.2	122.0	9.3	149.5	15.0	6.8
PLA/Cop-IA0.1	59.3	119.7	13.3	148.9	18.3	5.4
PLA/Cop-IA1	62.6	126.5	4.1	150.2	9.3	6.2
PLA/Cop-IA3	59.4	125.1	7.3	153.6	13.8	7.1
PLA/Cop-IA10	59.4	122.9	6.7	150.4	16.2	10.5
PLA/Net-IA0.1	60.3	119.6	13.2	152.3	19.4	6.7
PLA/Net-IA1	59.5	120.3	12.9	150.5	19.2	6.8
PLA/Net-IA3	59.6	120.6	10.6	151.2	19.1	9.4
PLA/Net-IA10	64.0	121.4	11.9	153.6	25.6	16.4

^1^ T_g_: glass transition temperature; ^2^ T_cc_: cold crystallization temperature; ^3^ ΔH_cc_: enthalpy cold crystallization; ^4^ T_m_: melting temperature; ^5^ ΔH_m_: melting enthalpy; ^6^ χ_cc_: crystallization grade.

**Table 3:** DMA result overview of PLA/IA, PLA/PIA, PLA/Cop-IA, and PLA/Net-IA blends.

Sample	T_g_ ^1^ (°C)	E’ max ^2^ (MPa)	σ max ^3^ (MPa)	ε ^4^ (%)
PLA	61.4	2733.3	32.6	3.4
PLA-IA0.1	64.6	657.6	30.6	41.6
PLA/IA1	61.3	5284.3	41.5	7.0
PLA-IA3	54.1	2642.6	37.3	3.7
PLA-IA10	58.2	1478.0	8.2	2.0
PLA/PIA0.1	62.9	1131.4	21.9	14.2
PLA/PIA1	62.9	872.6	28.5	17.6
PLA/PIA3	62.4	1086.8	24.4	3.2
PLA/PIA10	63.7	2769.4	32.7	6.3
PLA/Cop-IA0.1	64.4	3462.7	27.1	3.4
PLA/Cop-IA1	62.9	3271.6	28.6	3.1
PLA/Cop-IA3	62.8	4421.0	30.9	3.1
PLA/Cop-IA10	60.3	4200.0	35.3	3.8
PLA/Net-IA0.1	63.1	2005.1	29.6	3.0
PLA/Net-IA1	62.7	1887.5	24.5	3.2
PLA/Net-IA3	64.2	2889.2	22.1	3.0
PLA/Net-IA10	63.5	2735.7	21.2	2.7

^1^ T_g_: glass transition temperature. ^2^ E’: elastic modulus. ^3^ σ stress. ^4^ ε strain.

## Data Availability

The original contributions presented in the study are included in the article/Supplementary Material, further inquiries can be directed to the corresponding authors.

## Supplementary Materials

The following supporting information can be downloaded at https://www.mdpi.com/article/10.3390/polym16192780/s1; Figure S1: Infrared spectrum of poly(itaconic acid)-*co*-poly(methyl itaconate) (Cop-IA); Figure S2: SEM micrograph of poly(lactic acid)/itaconic acid (IA) 0.1 wt% (PLA/IA0.1) blend; Figure S3: SEM micrograph of poly(lactic acid)/itaconic acid (IA) 1 wt% (PLA/IA1) blend; Figure S4: SEM micrograph of poly(lactic acid)/itaconic acid (IA) 3 wt% (PLA/IA3) blend; Figure S5: SEM micrograph of poly(lactic acid)/itaconic acid (IA) 10 wt% (PLA/IA10) blend; Figure S6: SEM micrograph of poly(lactic acid)/poly(itaconic acid) (PIA) 0.1 wt% (PLA/PIA0.1) blend; Figure S7: SEM micrograph of poly(lactic acid)/poly(itaconic acid) (PIA) 3 wt% (PLA/PIA3) blend; Figure S8: Han plot curves of (a) PLA/IA, (b) PLA/PIA, (c) PLA/Cop-IA, and (d) PLA/Net/IA; Figure S9: DSC curves for poly(lactic acid)/itaconic acid blends; Figure S10: DSC curves for poly(lactic acid)/poly(itaconic acid) blends; Figure S11: DSC curves for poly(lactic acid)/(poly(itaconic acid)-co-poly(methyl itaconate)) blends; Figure S12: DSC curves for poly(lactic acid)/net-poly(itaconic acid)-*v*-triethylene glycol dimethacrylate blends; Figure S13: DSC curves (second heating) for PLA/Net-IA10 blend and its first derivative for determining enthalpy; Figure S14: X-ray diffraction for poly(lactic acid)/itaconic acid blends; Figure S15: X-ray diffraction for poly(lactic acid)/poly(itaconic acid) blends; Figure S16: X-ray diffraction for poly(lactic acid)/(poly(itaconic acid)-co-poly(methyl itaconate)) blends; Figure S17: X-ray diffraction for poly(lactic acid)/net-poly(itaconic acid)-*v*-triethylene glycol dimethacrylate blends; Figure S18: TGA, DTG, and DSC of itaconic acid; Table S1: Parameters utilized for Carreau–Yasuda model; Table S2: Comparison of T_g_, DMA vs. DSC; Table S3: Thermogravimetric analysis of PLA/IA, PLA/PIA, PLA/Cop-IA, and PLA/Net-IA blends; Equation (S1): Equation for Carreau–Yasuda model; Equation (S2): Equation for crystallinity degree.
