# Supplementary material for: Innovative Poly(lactic Acid) Blends: Exploring the Impact of the Diverse Chemical Architectures from Itaconic Acid
Source: Polymers (Basel). 2024 Sep 30;16(19):2780. doi: 10.3390/polym16192780 (PMC11478570; doi:10.3390/polym16192780)
Supplement: Supplementary file 1 [file polymers-16-02780-s001.zip › polymers-3133269-supplementary.pdf]

Article

Innovative PLA/Itaconic Acid Blends: Exploring the Impact of Chemical Architecture Variations

Miriam Carrasco-Fernández [1], Erika Ivonne López-Martínez [1], Sergio Gabriel Flores-Gallardo [1], Iván Alziri Estrada-Moreno [1], Mónica Elvira Mendoza-Duarte [1,*] and Alejandro Vega-Rios [1,*]

[1] Centro de Investigación en Materiales Avanzados, SC, Av. Miguel de Cervantes #120, Chihuahua 31136, México; e-mail@e-mail.com

* Correspondence: M.E.M.D. monica.mendoza@cimav.edu.mx; A.V.R. alejandro.vega@cimav.edu.mx; Tel.: +52614439483.

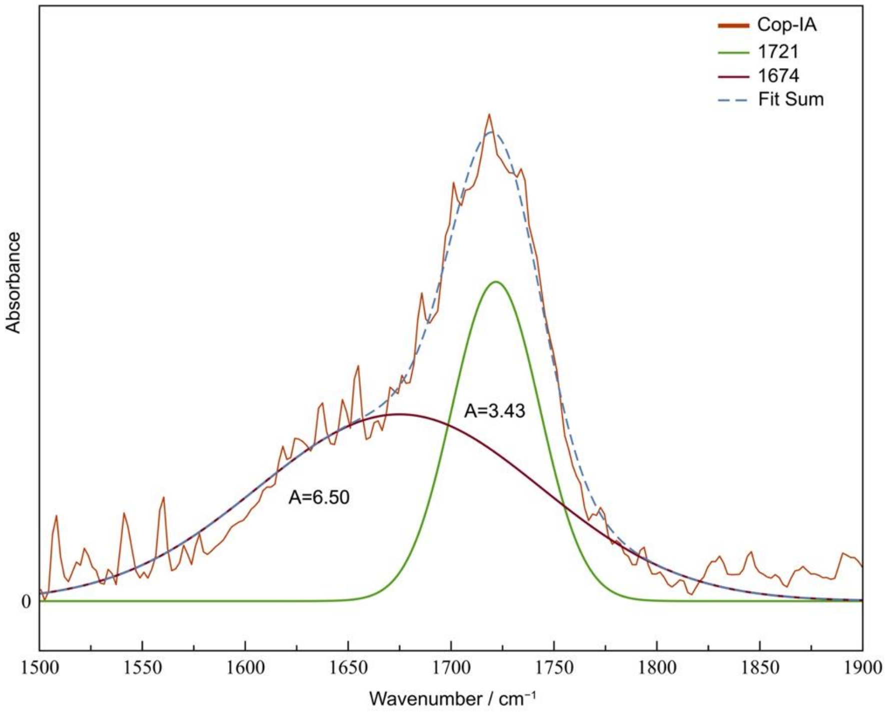

Figure S1. Infrared spectrum of poly(itaconic acid)-*co*-poly(methyl itaconate) (Cop-IA)

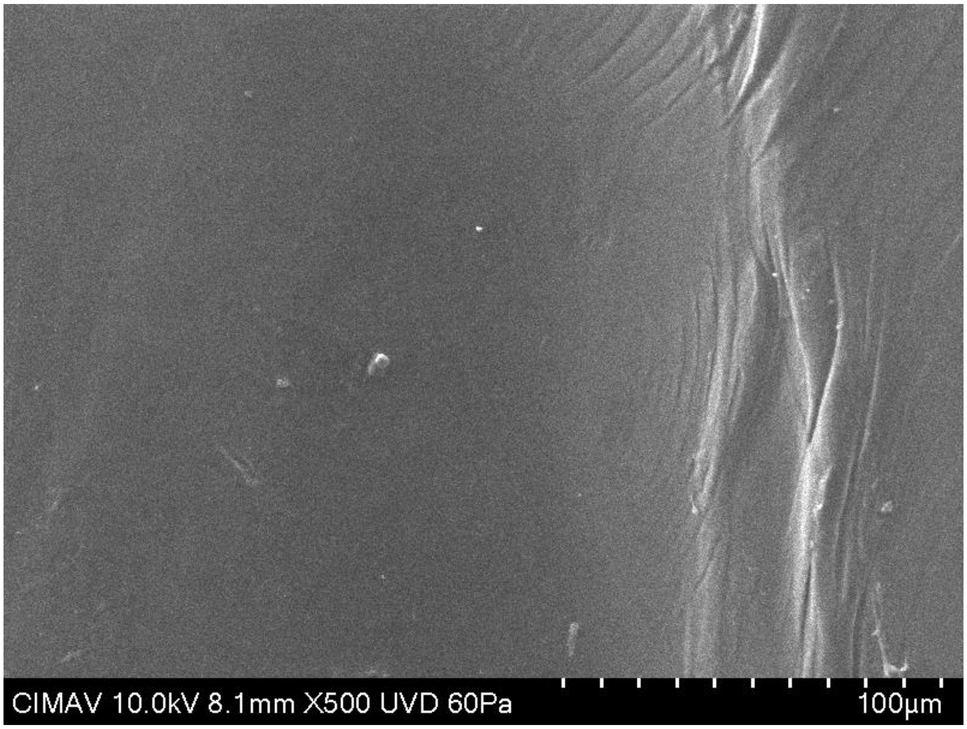

Figure S2. SEM micrograph poly(lactic acid) / itaconic acid (IA) 0.1 wt% (PLA/IA0.1) blend.

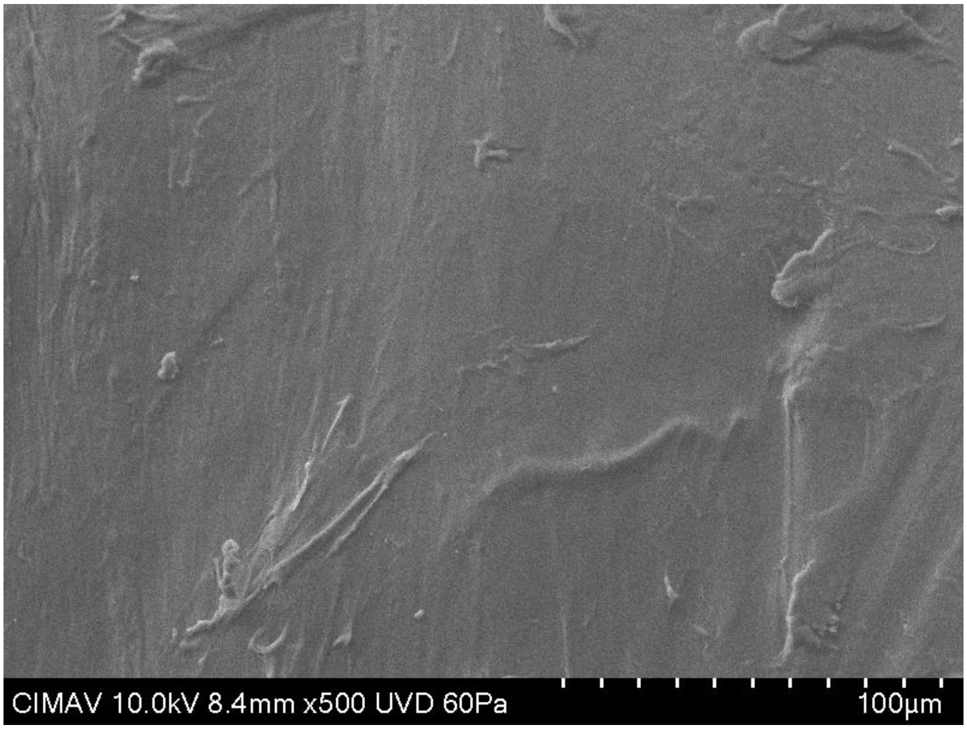

Figure S3. SEM micrograph poly(lactic acid) / itaconic acid (IA) 1 wt% (PLA/IA1) blend.

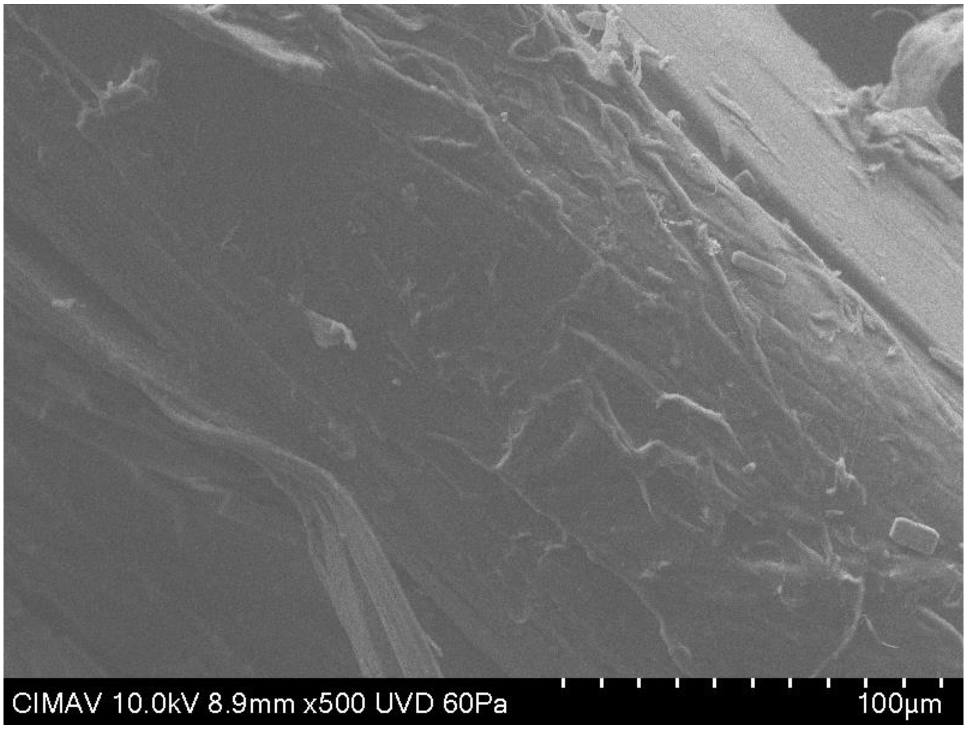

Figure S4. SEM micrograph poly(lactic acid) / itaconic acid (IA) 3 wt% (PLA/IA3) blend.

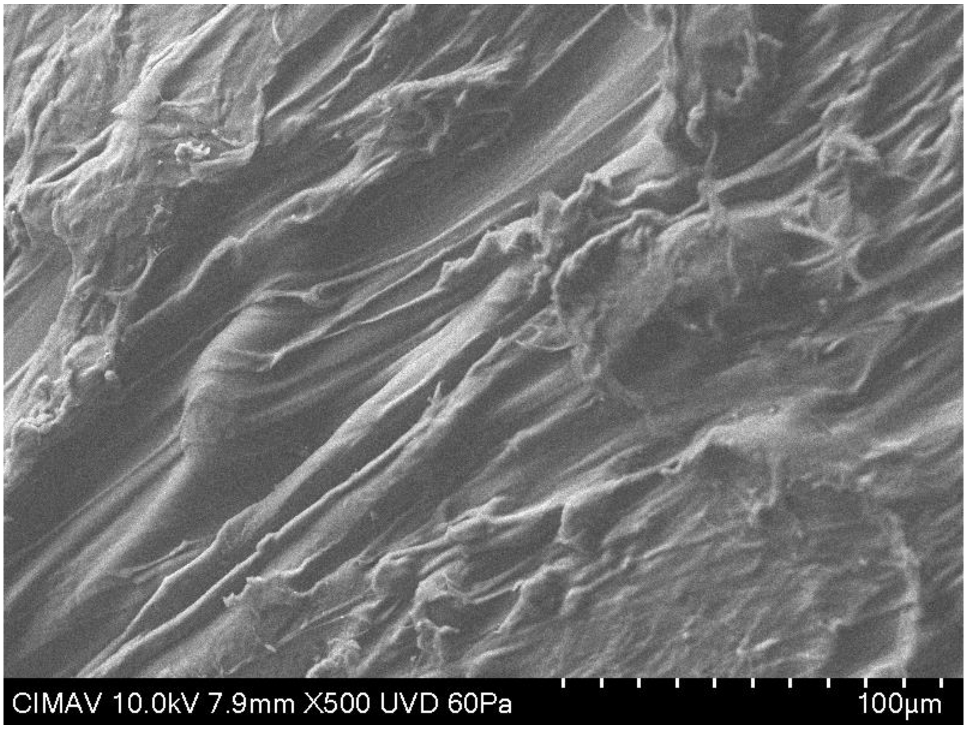

Figure S5. SEM micrograph poly(lactic acid) /poly(itaconic acid) (PIA) 0.1 wt% (PLA/PIA0.1) blend.

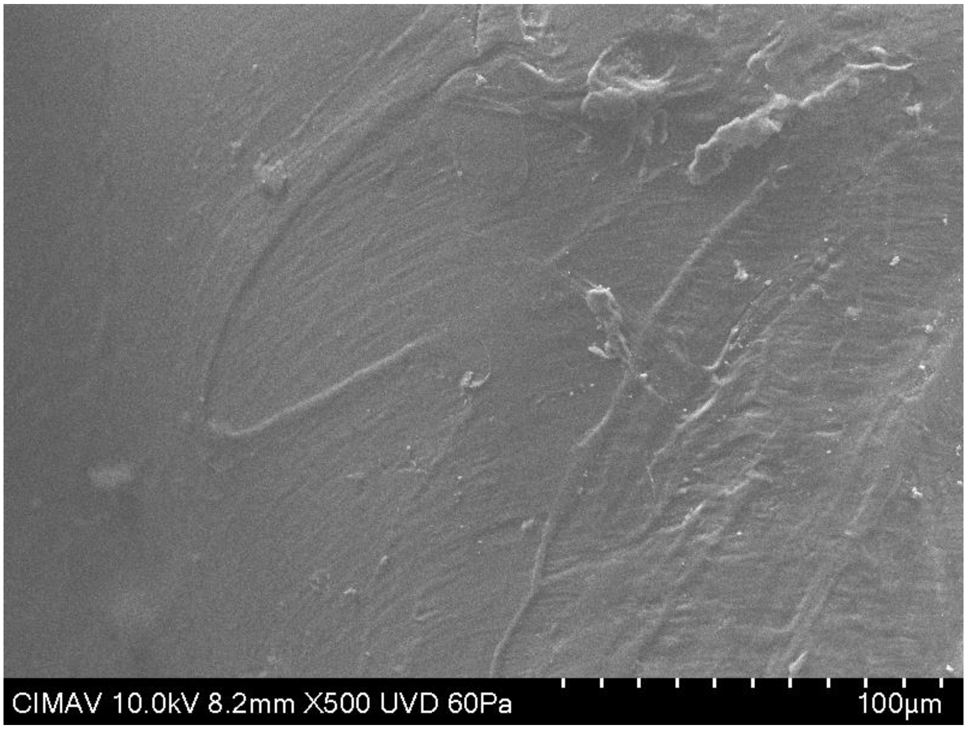

Figure S6. SEM micrograph poly(lactic acid) / poly(itaconic acid) (PIA) 1 wt% (PLA/PIA1) blend.

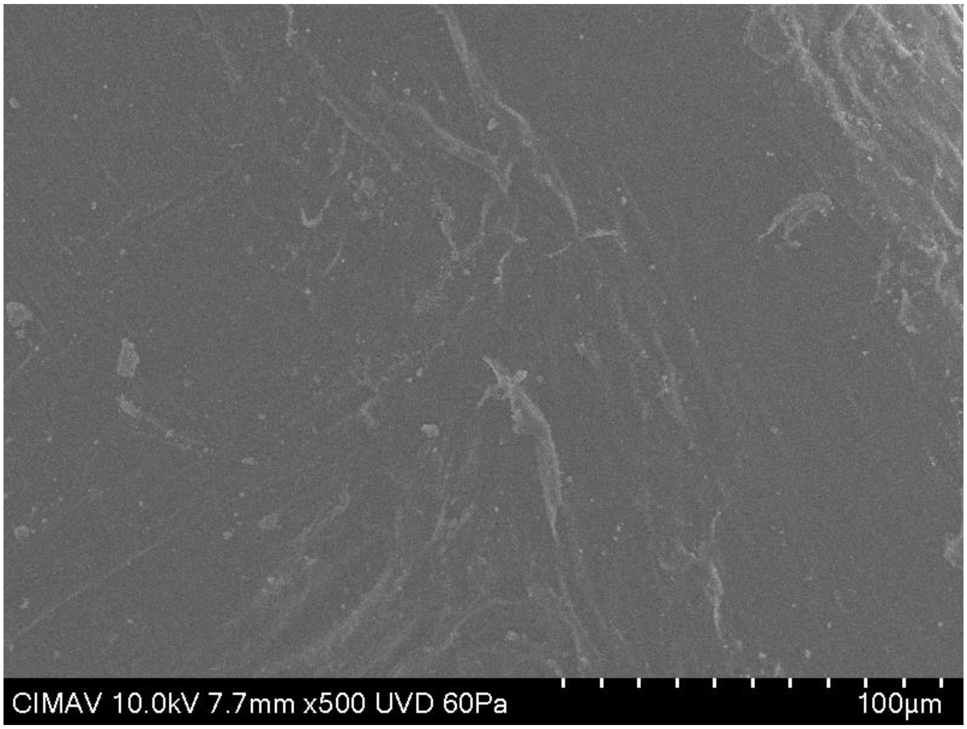

Figure S7. SEM micrograph poly(lactic acid) / poly(itaconic acid) (PIA) 3 wt% (PLA/PIA3) blend.

Table S1. Parameters utilized for the Carreau-Yasuda model.

Sample	η_0 (Pa.s)	λ (s)	n	a	R^2
PLA	2073.1	0.0249	0.3856	1.1138	0.9901
PLA/IA0.1	1999.3	0.0236	0.3444	1.0499	0.9951
PLA/IA1	1175.7	0.0167	0.3976	1.0932	0.9956
PLA/IA3	113.8	0.0073	0.7476	3.5359	0.6168
PLA/IA10	----	----	----	----	----
PLA/PIA0.1	3568.9	0.0454	0.3531	1.0338	0.9985
PLA/PIA1	4118.4	0.0514	0.3497	0.9509	0.9996
PLA/PIA3	3020.1	0.0342	0.3799	1.1595	0.9879
PLA/PIA10	2252.5	0.0307	0.4464	1.3638	0.9682
PLA/Cop-IA0.1	3417.1	0.0310	0.3254	0.9276	0.9964
PLA/Cop-IA1	3010.0	0.0314	0.3468	0.9793	0.9945
PLA/Cop-IA3	2806.1	0.0311	0.3699	1.0207	0.9929
PLA/Cop-IA10	3584.1	0.0298	0.3122	0.9131	0.9979
PLA/Net-IA0.1	3607.7	0.0253	0.2683	0.8424	0.9995
PLA/Net-IA1	2897.8	0.0315	0.3454	1.0231	0.9983
PLA/Net-IA3	3545.9	0.0279	0.2763	0.8596	0.9998
PLA/Net-IA10	858.7	0.0194	0.5202	2.1236	0.9219

Equation S1. Equation for the Carreau-Yasuda model.

$$\eta = \frac{\eta_0}{\left[1 + (\lambda\dot{\gamma})^a\right]^{\frac{1-n}{a}}}$$

Where:

η_0= zero shear viscosity (Pa.s).

λ= transition time (s).

a= regression parameter.

n= regression parameter.

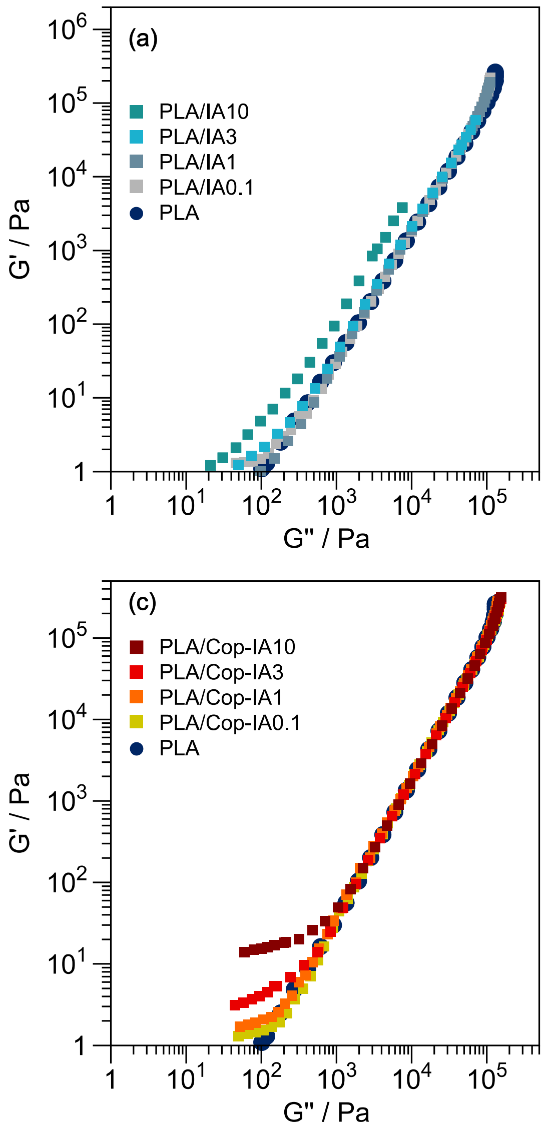

Figure S8. Han plot curves (a) PLA/IA; (b) PLA/PIA; (c) PLA/Cop-IA; (d) PLA/Net-IA.

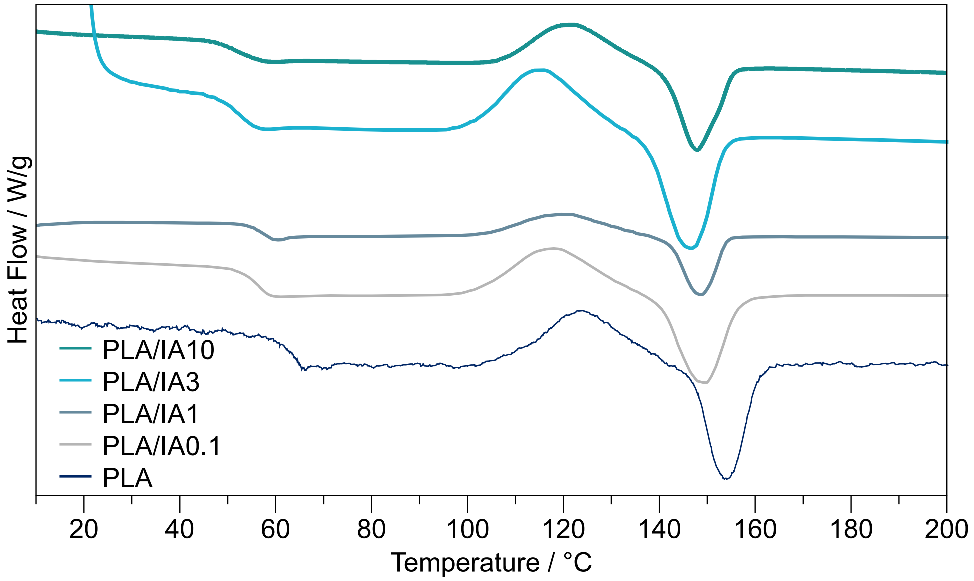

Figure S9. DSC curves for poly(lactic acid)/itaconic acid blends.

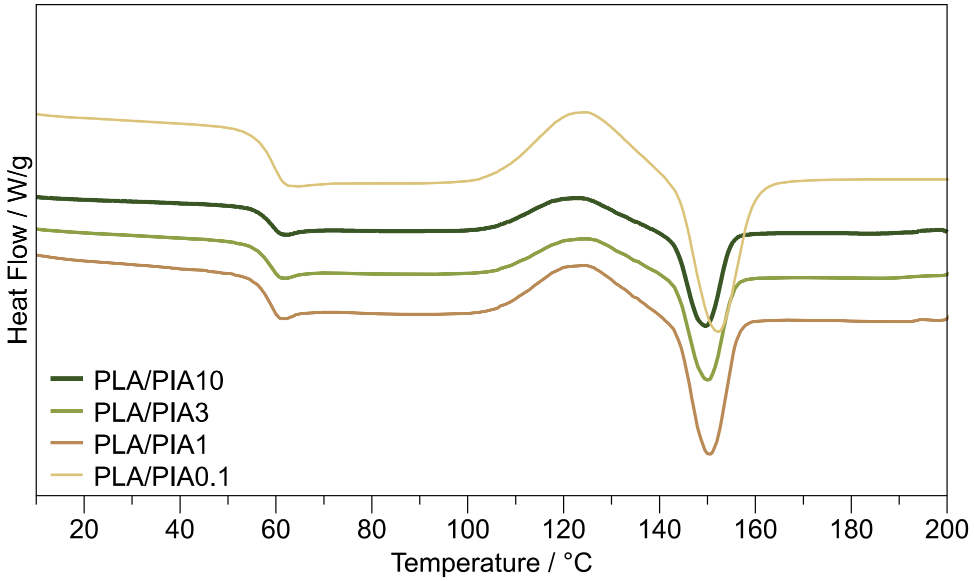

Figure S10. DSC curves for poly(lactic acid)/poly(itaconic acid) blends.

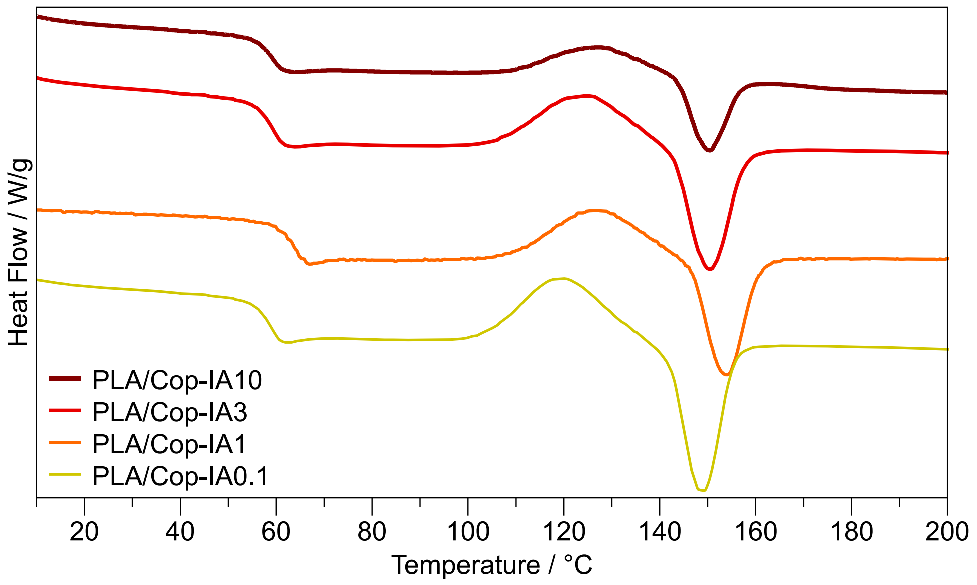

Figure S11. DSC curves poly(lactic acid)/(poly(itaconic acid)-*co*-poly(methyl itaconate)) blends.

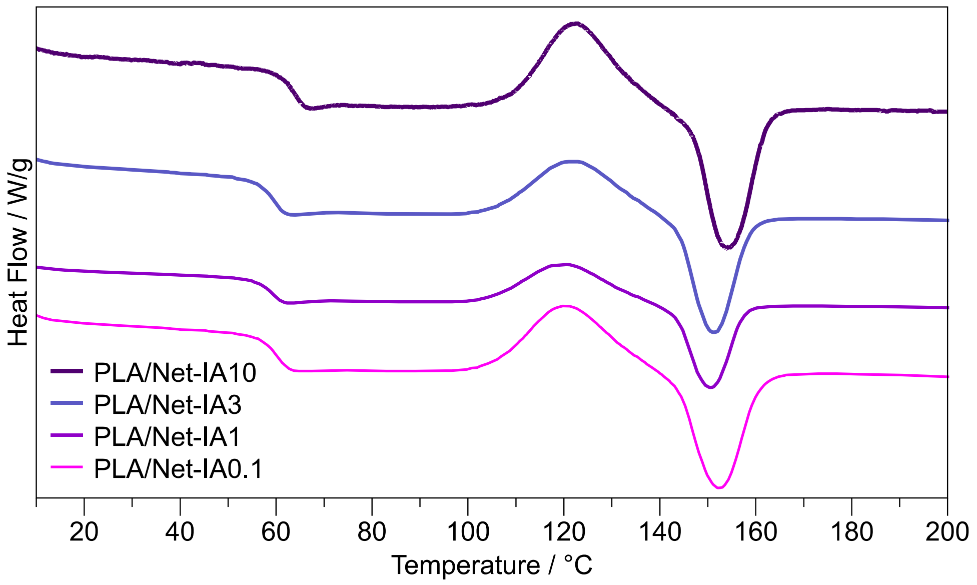

Figure S12. DSC curves for poly(lactic acid)/net-poly(itaconic acid)-ν-triethylene glycol dimethacrylate blends.

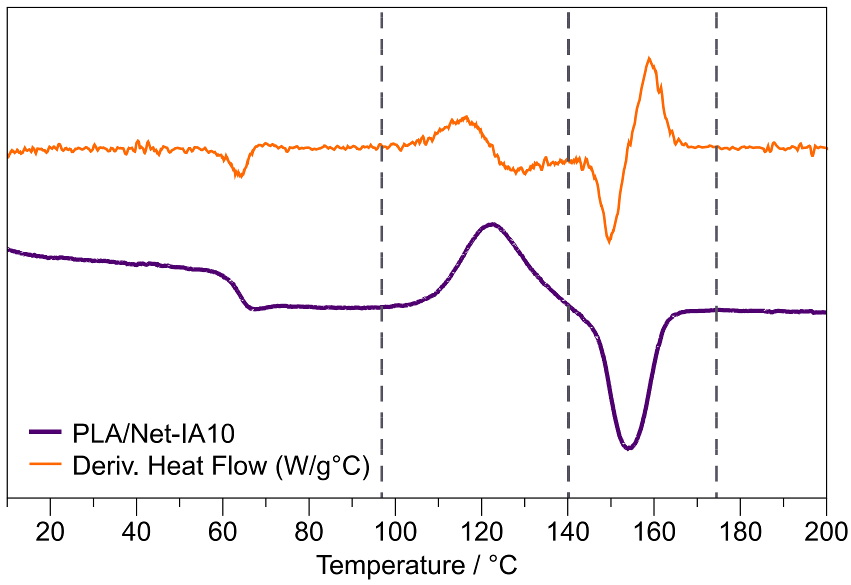

Figure S13. DSC curve (second heating) for PLA/Net-IA10 blend and its first derivative for determining enthalpy.

Equation S2. Equation for determined crystallinity degree.

$$X_{cc} = \left(\frac{\Delta H_m - \Delta H_{cc}}{W_f * \Delta H_{100\%c}} \right) * 100$$

Where:

ΔH_{cc}= cold crystallization enthalpy.

ΔH_m= fusion enthalpy.

$\Delta H_{100\%c}$= enthalpy assuming of 100% crystalline PLA (93 J/g).

W_f= weight fraction of the PLA in the blend.

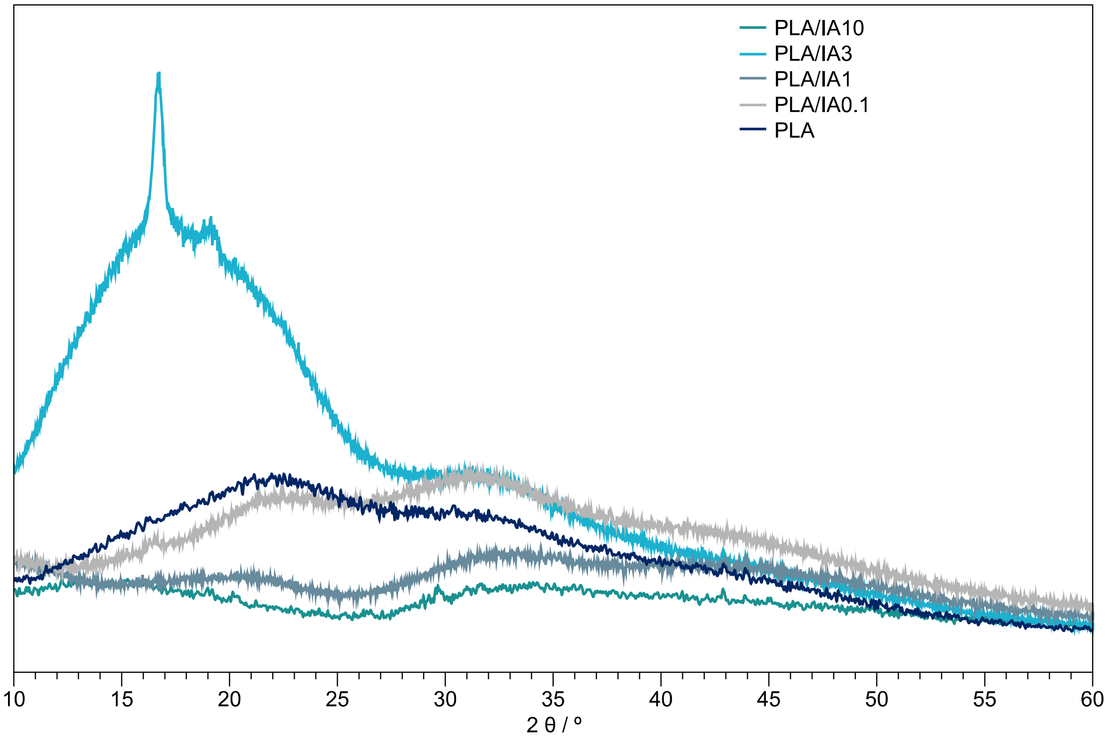

Figure S14. X-ray diffraction for poly(lactic acid)/itaconic acid blends.

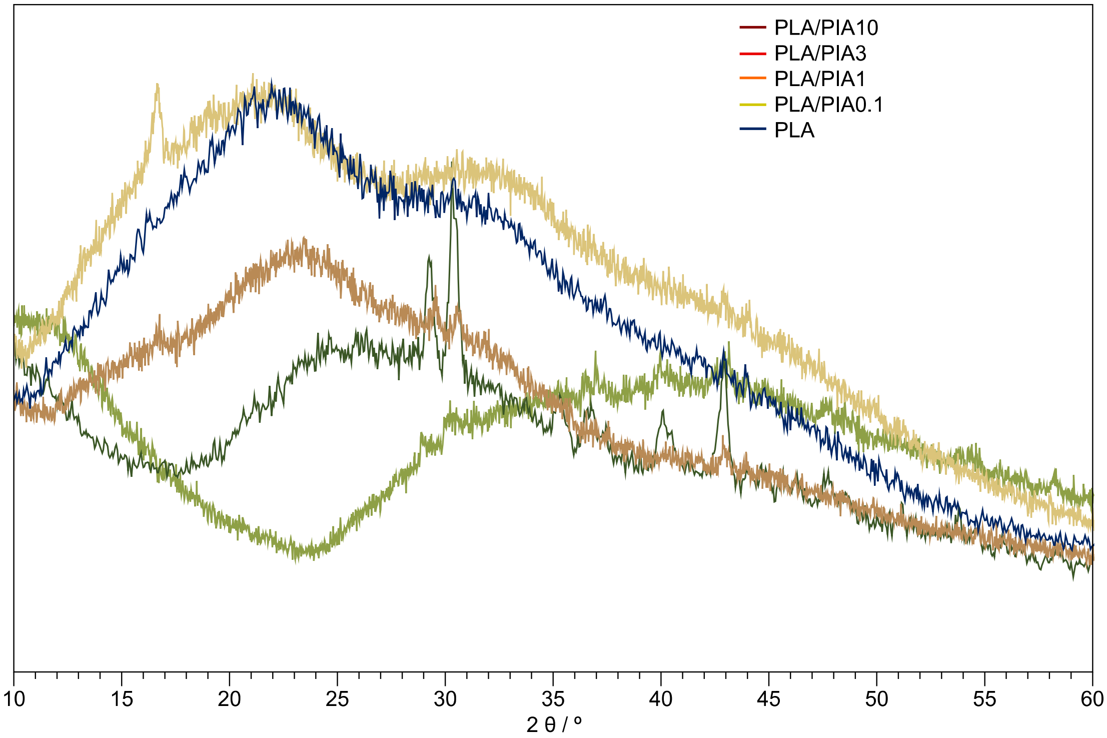

Figure S15. X-ray diffraction for poly(lactic acid)/poly(itaconic acid) blends.

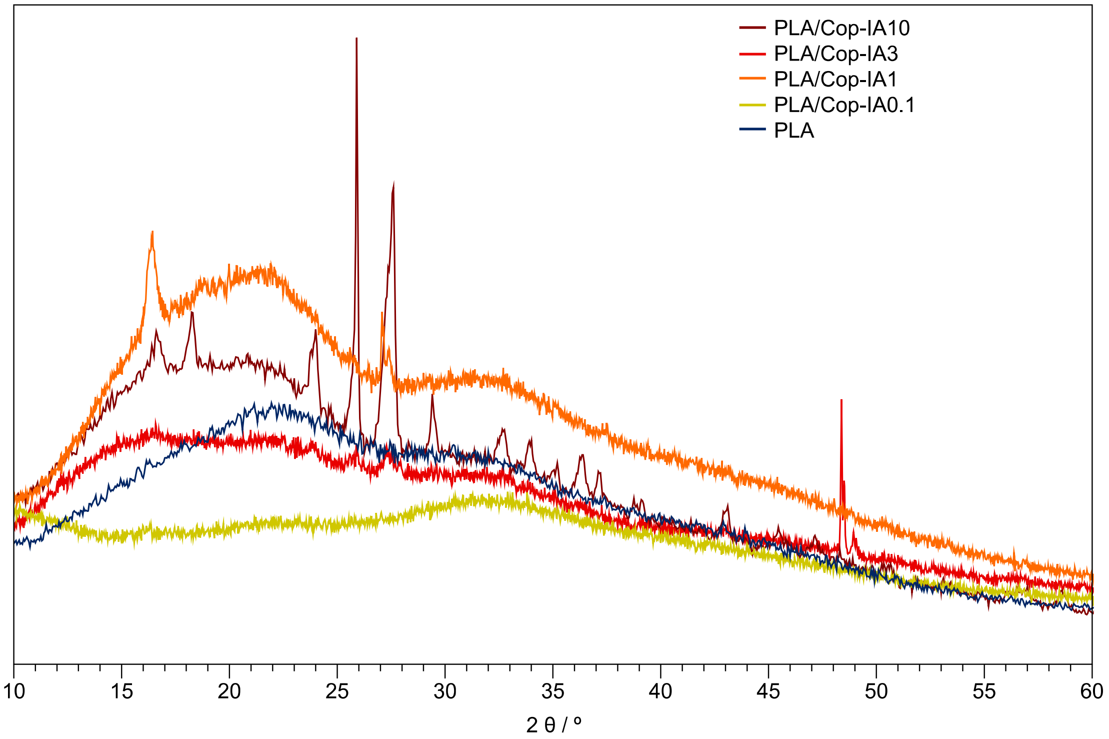

Figure S16. X-ray diffraction for poly(lactic acid)/(poly(itaconic acid)-*co*-poly(methyl itaconate)) blends.

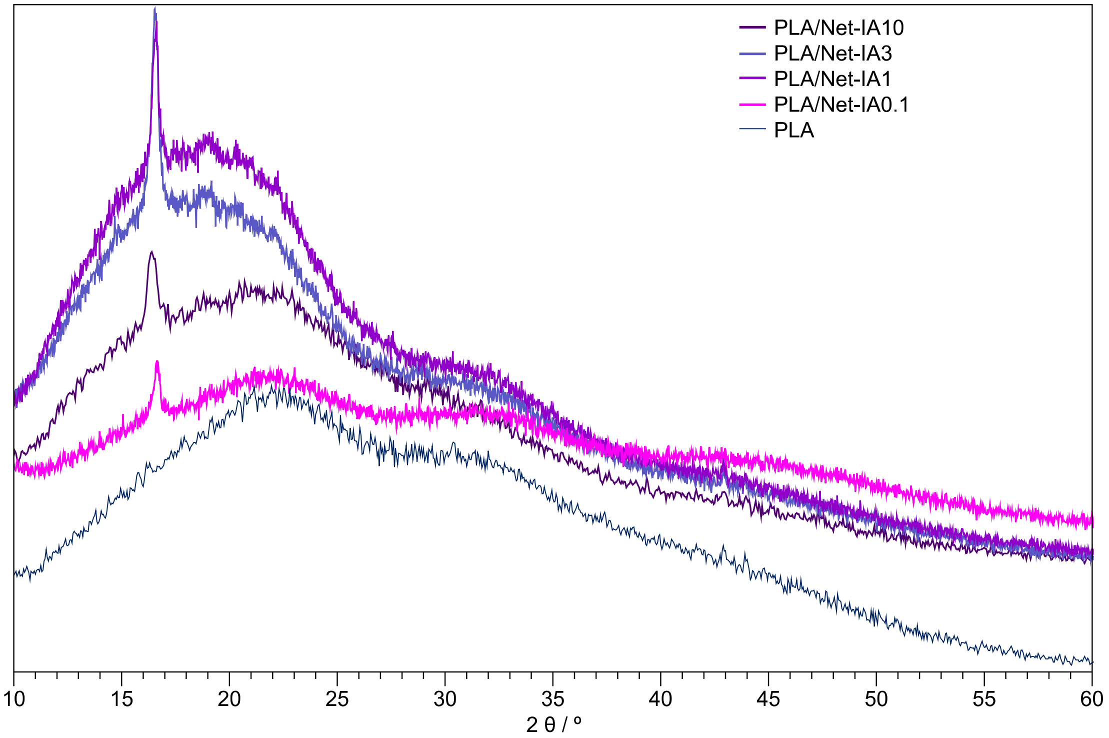

Figure S17. X-ray diffraction for poly(lactic acid)/net-poly(itaconic acid)-ν-triethylene glycol dimethacrylate blends.

Table S2. Comparison of T_g, DMA *vs* DSC.

Sample	T_g DMA (°C)	T_g DSC (°C)
PLA	61.4	65.2
PLA/IA0.1	64.6	56.6
PLA/IA1	61.3	58.5
PLA/IA3	54.1	49.0
PLA/IA10	58.2	51.2
PLA/PIA0.1	62.9	59.6
PLA/PIA1	62.9	59.3
PLA/PIA3	62.4	59.1
PLA/PIA10	63.7	59.2
PLA/Cop-IA0.1	64.4	59.3
PLA/Cop-IA1	62.9	62.6
PLA/Cop-IA3	62.8	59.4
PLA/Cop-IA10	60.3	59.4
PLA/net-IA0.1	63.1	60.3
PLA/net-IA1	62.7	59.5
PLA/net-IA3	64.2	59.6
PLA/net-IA10	63.5	64.0

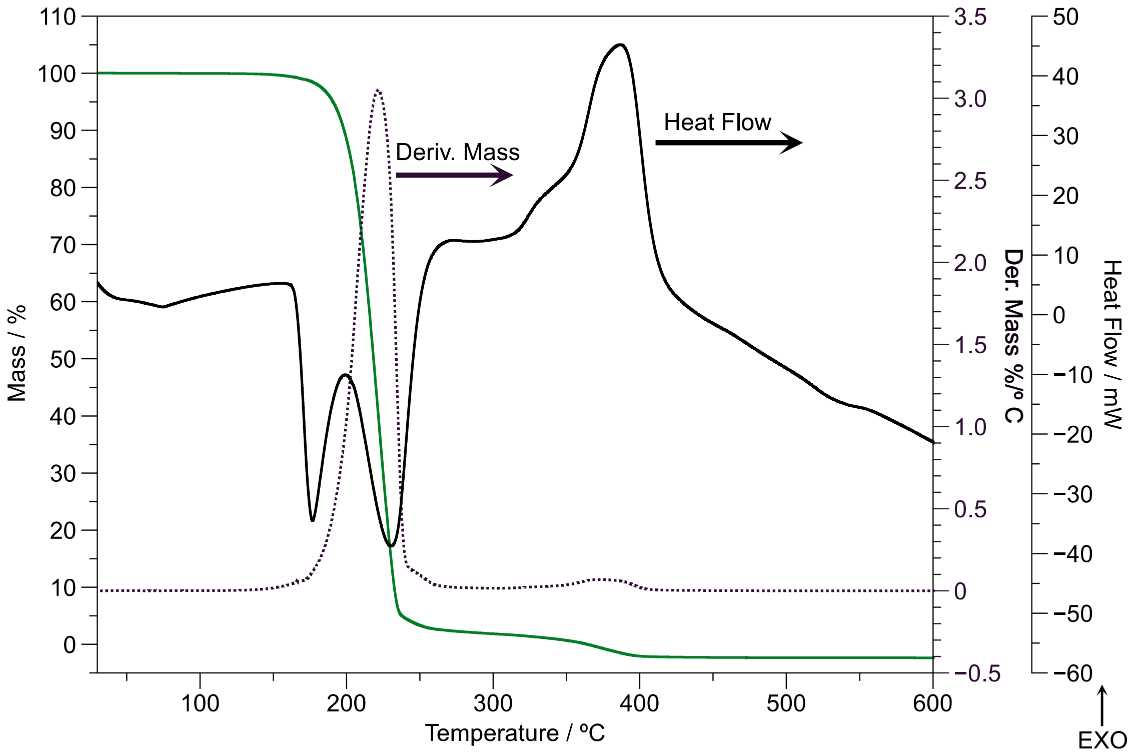

Figure S18. TGA, DTG and DSC of itaconic acid.

Table S3. Thermogravimetric analysis of PLA/IA, PLA/PIA, PLA/Cop-IA, and PLA/Net-IA blends

Sample	T_5 (°C)	Variance (%)	T_{10} (°C)	Variance (%)	T_{50} (°C)	Variance (%)	T_{max} (°C)	Variance (%)
PLA	321.3	0.54	331.8	0.28	355.3	1.23	359.1	1.26
PLA/IA0.1	320.1	3.11	333.1	4.54	357.6	0.81	361.0	4.87
PLA/IA1	321.9	2.00	331.6	3.33	358.6	2.37	360.6	1.29
PLA/IA3	281.9	1.68	315.5	1.50	351.8	2.41	361.9	1.87
PLA/IA10	259.2	1.73	303.2	6.03	358.4	6.31	364.2	2.37
PLA/PIA0.1	328.0	4.37	336.7	1.04	360.4	5.74	363.0	3.35
PLA/PIA1	330.8	4.81	339.5	5.06	363.0	3.07	371.3	6.82
PLA/PIA3	329.6	2.48	339.4	3.66	362.2	2.37	363.3	7.35
PLA/PIA10	328.9	1.14	336.7	1.64	355.6	1.28	359.5	2.02
PLA/Cop-IA0.1	329.9	5.48	338.3	3.01	359.2	3.60	369.3	1.62
PLA/Cop-IA1	327.5	1.47	339.2	2.20	358.5	4.99	364.1	3.07
PLA/Cop-IA3	330.3	3.08	339.0	5.00	362.7	3.22	364.6	5.00
PLA/Cop-IA10	330.9	4.49	339.9	1.03	361.0	5.73	371.3	1.84
PLA/Net-IA0.1	329.9	6.77	325.8	2.57	356.3	4.16	366.6	5.87
PLA/Net-IA1	324.3	1.58	336.3	6.80	355.4	3.78	362.7	2.11
PLA/Net-IA3	326.8	3.60	335.0	3.00	359.9	3.01	362.7	4.21
PLA/Net-IA10	333.8	4.23	341.6	2.09	360.6	2.37	363.7	5.00
